# Design and development of a portable multiwavelength LED-based diffuse reflectance spectroscopy tool for rapid breast cancer identification

**DOI:** 10.1038/s41598-025-25867-8

**Published:** 2025-11-25

**Authors:** Arif Mohd. Kamal, Kirteyman Singh Rajput, Apurva Dahake, Gayatri Gogoi, Ajay Krishnan Ajith, V. S. N. Sitaramgupta Vangeti, Dilip Kiling, Jayant S. Vaidya, Hardik J. Pandya

**Affiliations:** 1 Department of Electronic Systems Engineering, Indian Institute of Science, Bangalore, India; 2 Medical Research Unit Laboratory, Department of Pathology, Assam Medical College, Dibrugarh, India; 3 Department of Surgery, Assam Medical College, Dibrugarh, India; 4 Division of Surgery and Interventional Science, University College London, London, UK; 5 Department of Design and Manufacturing, Indian Institute of Science, Bangalore, India

**Keywords:** Breast cancer, Near-infrared spectroscopy, Biomedical engineering, Breast cancer

## Abstract

Breast cancer is the most prevalent cancer among women worldwide, emphasizing the need for rapid and accurate diagnostic tools to improve patient outcomes and survival rates. In this study, we developed a diagnostic tool—a multispectral pen based on diffuse reflectance spectroscopy (DRS)—to enable real-time ex vivo differentiation between malignant and adjacent normal human breast tissues, primarily based on lipid and collagen absorbers. Diffuse reflectance was observed to be higher, while reduced absorbance was lower for malignant tissue compared to adjacent normal tissue across 62 samples (50 formalin-fixed and 12 fresh tissues). Clinical data from 31 patients, with paired adjacent normal and malignant samples per patient, revealed significantly lower mean reduced absorbance values for malignant tissue at three wavelengths: 850 nm (0.13 ± 0.02 vs. 0.28 ± 0.02), 940 nm (0.19 ± 0.01 vs. 0.37 ± 0.02), and 1050 nm (0.25 ± 0.03 vs. 0.43 ± 0.03) with *p* < 0.0001 across formalin-fixed tissue samples. Using a CatBoost machine learning model, the tool achieved an accuracy of 90%, a sensitivity of 80%, and a specificity of 100% in distinguishing malignant from normal tissues. Limitations include a small sample size and limited patient diversity. Future work aims to integrate the DRS technology with machine learning algorithms to provide real-time intraoperative margin assessment, and testing in a larger study.

**Supplementary Information:**

The online version contains supplementary material available at 10.1038/s41598-025-25867-8.

## Introduction

Breast cancer remains the most common cancer among women globally, with an estimated 2.3 million new cases and 670,000 deaths reported worldwide, as per the recent reports published by the International Agency for Research on Cancer (IARC) and the World Health Organization (WHO). Projections suggest that by 2050, the incidence rate of breast cancer cases will increase by 38%, and the death rate will rise by 63% worldwide^1,2^. Despite this burden, breast cancer exemplifies how early detection and advances in therapy can improve outcomes.

The diagnosis of breast cancer typically follows a standardized triple-assessment approach comprising clinical evaluation, imaging, and histopathological confirmation. The clinical assessment involves a detailed patient history and physical examination to identify palpable lumps or associated symptoms. Several imaging techniques are used to screen and diagnose breast cancer^3^, with X-ray mammography being the most popular and successful screening method. But overall, its sensitivity is moderate (about 75%), and in women with mammographically dense breast tissues, it falls to 62%^4^. Furthermore, X-ray mammography has a 22% false-positive rate in women under the age of fifty^5^ and has trouble accurately distinguishing between benign and malignant tumors^6^. At times, mammography is performed in conjunction with complementary imaging techniques like ultrasonography^7,8^ and magnetic resonance imaging (MRI)^8,9^. Both techniques, however, have drawbacks: ultrasound has comparatively low sensitivity, while MRI is costly and has restricted throughput and specificity. Non-invasive diagnostic techniques are often followed by a tissue needle biopsy, an invasive procedure to collect 4 μm-thick samples from the region of interest. These samples undergo histopathological examination involving slicing, staining with Hematoxylin and Eosin (H&E), and microscopic evaluation. The tumor is resected from the body, as part of surgical treatment, by performing either a lumpectomy (breast-conserving surgery (BCS)) or a mastectomy^10^, with or without axillary lymph node detection, based on the stage of the disease. After the surgery, the complete tumor resection is confirmed by performing histopathology on smaller sections of the resected region. If the histopathology results show that tumor is not completely resected (i.e., present at the margin of excision), and then in that scenario, there is a high risk of relapse and recurrence. The patient might be required to undergo surgery again^10^. Second surgeries in breast cancer can delay adjuvant therapy, which is ideally initiated within 4–6 weeks after the final operation. Evidence shows that each 4-week delay increases recurrence and mortality risk, with delays beyond 12 weeks markedly worsening outcomes. The adverse impact is most pronounced in HER2-positive and triple-negative breast cancers, where even modest postponements beyond 6 weeks significantly reduce survival. International guidelines (e.g., ESMO) stress minimizing such delays through proactive surgical and treatment planning^11,12^. The rate at which a second surgery (re-excision) is needed to clear margins after initial breast-conserving surgery for breast cancer typically ranges from 10% to 21%. This means about 1 in 10 to 1 in 5 patients require a second operation to achieve clear (negative) margins. Some studies report ranges from 10% to as high as 30%, depending on factors like tumor type (e.g., ductal carcinoma in situ has higher rates), age, tumor size, and institutional practices^13,14^. Effective cancer margin assessment is crucial to improve the 5-year survival rate among patients undergoing breast cancer surgery^15^. Furthermore, assessment of the needle core biopsy needs specialist histopathologist, a scare human resource. If a biopsy can be flagged in the field using a novel ‘pen’ then further management could be greatly expedited.

Optical imaging and spectroscopy in the near-infrared (NIR) region emerge as a transparent optical window for medical imaging^16^. This “optical window” allows for deep tissue penetration with minimal absorption from endogenous chromophores such as water and hemoglobin, enabling effective imaging and spectroscopy for various in vivo clinical applications^17,18^. Optical spectroscopy-based probes are generally simpler, portable, and easier to deploy. OS-based probes typically focus on a small range of wavelengths to balance cost-efficiency and clinical practicality. A few prior works discussed here have explored diffuse reflectance spectroscopy (DRS)-based studies. Shalini et al.^19^ developed a spectrometer to study the impairment of thyroid tissues after shining a light in the 700–1040 nm wavelength range to evaluate the functionality of thyroid glands. Nogueira et al.^20^ illustrated the use of DRS to detect colorectal cancer, where a tungsten halogen lamp with a wavelength range of 340 nm to 2400 nm shines light through an optical fiber onto the tissue samples. The limitation of this approach is the use of a lamp and a spectrometer, which consumes a lot of space and is not compatible with in vivo settings. Feder et al.^21^ proposed a novel methodology to remove the dependencies of the system on the geometry of photodetectors while assessing the oxygenation level of the index finger; their method was based on an iso-pathlength point using a single wavelength (i.e., 632 nm) laser source. Aref et al. reported a hyperspectral imaging-based system offering the ability to detect and delineate the margins of breast cancer in the tissues being analyzed in the wavelength range of (390–980 nm)^22^. Guergan et al.^23^ explored optical emission spectroscopy (OES) as a real-time method for identifying malignant breast tissue during surgery. By analyzing spectroscopic features from 972 tissue spectra using a support vector machine algorithm, it achieves an impressive classification accuracy of 96.9%, with high sensitivity, specificity, and predictive values. In another work, Aref et al.^24^ reported a visible range multiwavelength (i.e., 415 nm, 565 nm, 660 nm) optical imaging system using a CCD camera to highlight the difference in the reflectance values obtained from the malignant/normal excised breast tissue using the K-means clustering algorithm. Pal et al.^25^ showcased a multiwavelength portable platform to understand the absorption (primarily lipid and collagen) properties of the excised breast tissue in the transmission configuration. Higher absorption contrast factor and volume fraction contrast were observed in malignant breast tissues compared to adjacent normal tissues. To distinguish between malignant and adjacent normal breast biopsy tissues ex vivo, Kamal et al. ^26^ proposed a dual-wavelength (i.e., 850 nm and 940 nm) LED-based optical tool based on the time-domain NIRS method to quantify the effective attenuation coefficient of the excised breast biopsy tissues. Boer et al.^27^ reported a DRS technology-based biopsy needle for breast cancer margin assessment on excised lumpectomy from *N* = 16 patients that showed the fat/water ratio can be a potential biomarker for delineating cancer from adjacent normal tissues with a sensitivity and specificity of 100%. Using a cross-polarised micro-camera setup, Stefan D van der Stel. *et* al. demonstrated the real-time detection of microcalcification in the resected margin of superficial breast tissues. A depth of 2 mm was optically distinct from the adjacent normal tissue with an overall accuracy of 79% and specificity of 80%^28^. Hao Wang. *et* al. used a customised array of LEDs of 26 different wavelengths and a sensitive CMOS camera for margin assessment. Based on the field of view, the MSI algorithm tool identified the boundaries and directly projected them onto the BCS surgical cavity^29^. While significant research exists on margin assessment in general, a closer examination reveals that most methods are not truly real-time, are applied ex vivo, or are not portable and/or costly.

Spectroscopy analysis using machine learning (ML) methods can provide a fast, efficient, and reliable way to diagnose breast cancer effectively^30,31^. Srinivasu P.N. et al. reported a novel hybrid ML model, combining the CatBoost algorithm with a multi-layer perceptron neural network (CatBoost + MLP), aimed at improving breast cancer diagnosis. It emphasizes the importance of feature engineering using ANOVA and explainable AI techniques, such as SHAP values for interpretability. The model is tested on the Breast Cancer Wisconsin dataset, showcasing superior performance compared to conventional methods, achieving an accuracy of 99.3%. Comparative analysis with other models highlights its precision and efficiency in early diagnosis, aiding in personalized treatment approaches^32^.

This work presents an automated multiwavelength (i.e., 850 nm, 940 nm, and 1050 nm) LED-based multispectral pen for characterizing malignant and adjacent normal breast tissues ex vivo. The multispectral pen can estimate reduced absorbance (A_CWS_) using reflected light based on the collagen and lipid composition inside the tissue and further classify malignant tissues from the adjacent normal with the help of a ML model. By leveraging the capabilities of ML, this study contributes to improved diagnostic accuracy and fosters an affordable healthcare system.

The study is organized into the following sections: The methods and materials section describes tissue sample preparation, different modules of the multispectral pen, and the theoretical and classification model. The result and discussion section covers the experimental results, a discussion about the performance of the multispectral pen, a brief comparison with the other gold standard techniques, and limitations. Finally, the conclusion summarizes the findings and outlines the future research directions.

## Materials and methods

**Fig. 1:**
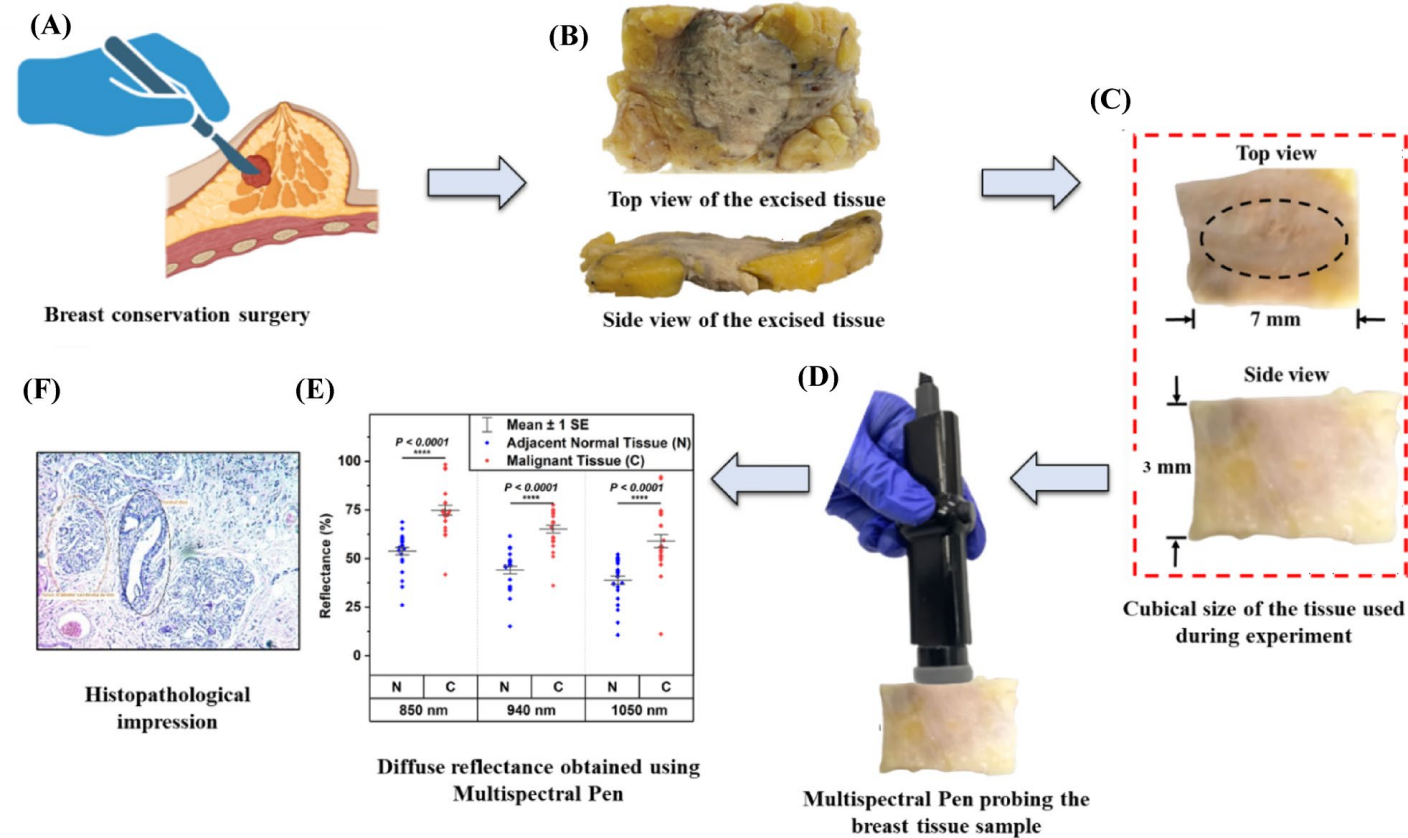
Conceptual diagnosis workflow of breast cancer diagnosis: (**A**) Conceptual representation of breast conservation surgery, (**B**) Actual image of the excised breast tissue during surgery, (**C**) Block of the breast tissue samples used during experiments, (**D**) Actual image of the multispectral pen on the tissue sample, (**E**) Diffuse reflectance results obtained using multispectral pen, and (**F**) Histopathological impression of a breast tissue sample.

This section explains the breast tissue sample preparation protocol, experimental procedure, mechanical, electrical, and optical modules associated with the multispectral pen, along with theoretical understanding and the ML-based classification model. The conceptual diagnosis workflow for excising breast tissue samples and their results obtained using the multispectral pen is shown in Fig. 1. The schematic of surgical intervention during BCS is shown in Fig. 1A, and the excised breast tissue’s top and side views are shown in Fig. 1B. Further, a tissue chunk resected from the excised breast tissue sample for the measurement is shown in Fig. 1C. The placement of the multispectral pen over the tissue sample is shown in Fig. 1D. The diffuse reflection plot obtained using the pen is shown in Fig. 1E, and a 10x zoomed image of a histopathological impression is shown in Fig. 1F.

### Preparation of tissue samples

The malignant tissue was excised along with adjacent normal tissue from the patient during BCS and kept in saline solution for fresh tissue study. After performing the study on fresh tissue samples, it was kept in a 10% formalin solution for 24 h for formalin fixation. The institutional ethics committee of Indian Institute of Science and Assam Medical College approved the methods and all experimental protocols. The patient’s written informed consent was obtained from all the subjects in accordance with the protocol submitted to the institutional ethics committee of the Indian Institute of Science and Assam Medical College, bearing ethical clearance certificate numbers 17-14012020 and AMC/EC/311. The malignancy and normalcy of the tissue samples were validated using the histopathology examination after the measurements were performed. The clinical diagnosis and histopathological impressions are shown in Supplementary Table 1. Before each measurement, the tissue was placed on the glass slide for 2 min at room temperature (24 °C). Afterwards, the tissue was placed on the black-colored sheet during the data acquisition to avoid any back reflection from the glass slides.

### Preparation and calibration of barium sulfate reflectance standard

Barium sulfate (BaSO₄) has been widely utilized as a low-cost, high-reflectance material for various optical applications, including its use as a reflectance standard and for coating integrating spheres. The material exhibits high diffuse reflectance properties in the wavelength range of 200–2000 nm, making it suitable as a reflectance standard for various applications^33^. The following section discusses the preparation and calibration of a sintered BaSO_4_ pellet used as a reflectance standard in this study.

The sintered barium sulfate (BaSO₄) reflectance standard was prepared from high-purity powder (≥ 99.9%, trace-metal grade) pressed and baked at 500 °C for 10 h. The dried pellet was sintered at 970 °C for 2 h to enhance its mechanical stability. After sintering, surface grinding and polishing were performed using high-grade sandpaper to achieve a uniform surface and to remove any globules present after the sintering^34^. The final sintered BaSO₄ pellet has a diameter of 23.5 mm with a thickness of 5.5 mm. It exhibits relative reflectance of 82.78%, 83.67%, and 85.23% at 850 nm, 940 nm, and 1050 nm, respectively and an average relative reflectance of 82.56 ± 1.86% (mean ± standard deviation) across the therapeutic window (600–1100 nm), as calibrated against a commercial reflectance standard showing suitability for biomedical and optical applications as a stable diffuse reflectance references in NIR region. All the experiments were performed using the available in-house reflectance standard. Later, the in-house standard was calibrated with respect to the commercial reflectance standard (SRS-99-010, Labsphere, USA) for any variability in the measurements.

**Fig. 2:**
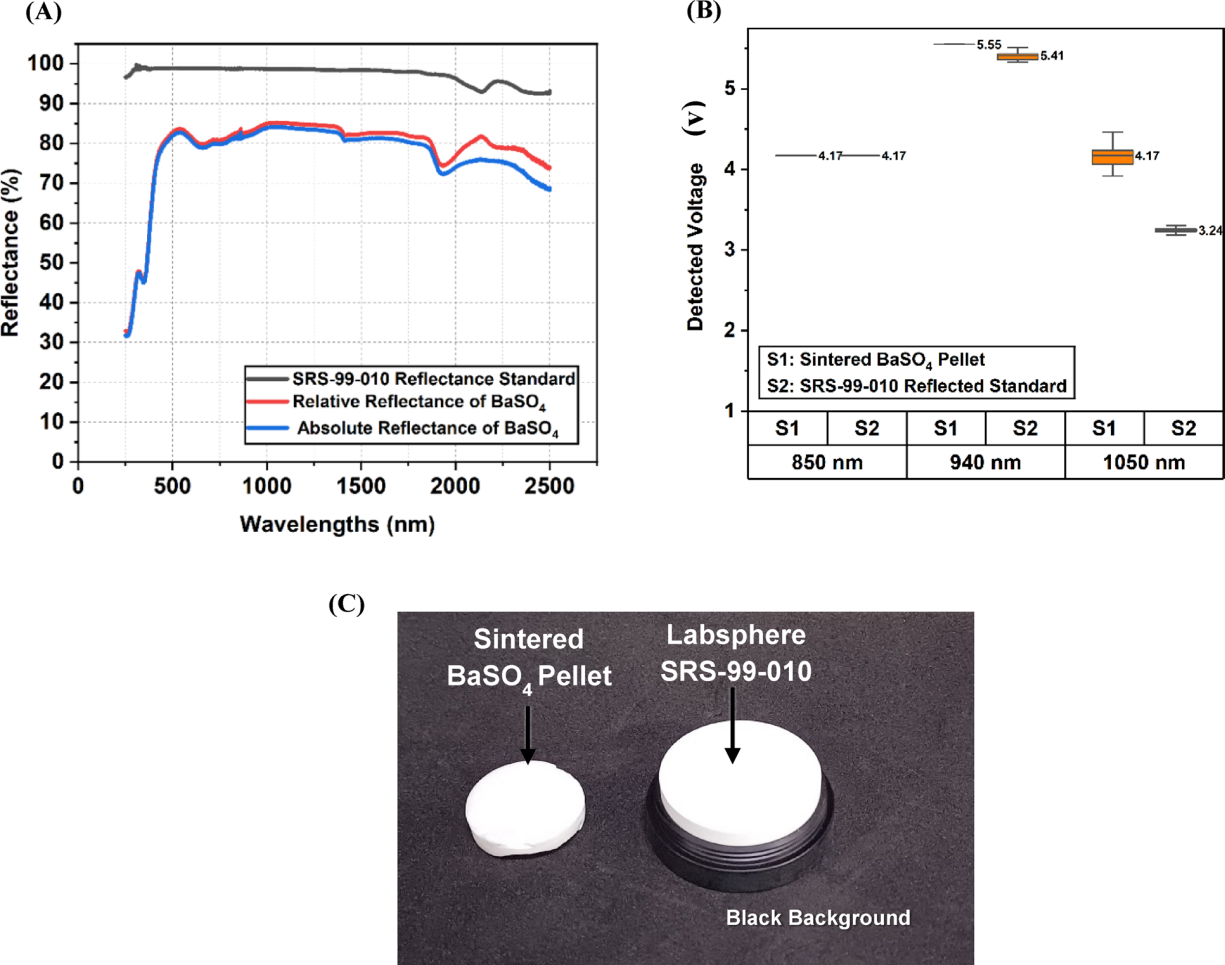
Comparison of in-house built reflectance standard (i.e., sintered BaSO_4_ pellet) with commercial reflectance standard (SRS-99-010, Labsphere, North Sutton, NH, USA): (**A**) Reflectance (%) measurements, (**B**) Detected voltage (V) measurements comparison, and (**C**) Image of in-house built sintered BaSO_4_ pellet and SRS-99-010 standard with black background.

The *SRS-99-010* is a commercially available polytetrafluoroethylene (PTFE)-based reflectance standard. Figure 2A shows the reflectance (%) plot of the *SRS-99-010* reflectance standard, relative and absolute reflectance of sintered BaSO_4_ reflectance standard for the wavelength range of (250–2500 nm) measured using a PerkinElmer Lambda 750 UV/Vis/NIR spectrophotometer. The spectrophotometer has an integrating sphere of a diameter of 60 mm, a sample port diameter of 19 mm, and a wall reflectance between 95 and 99%. Due to water absorption within the BaSO_4_, the spectrum also exhibits downward peaks near 1450 nm, 1950 nm, and 2500 nm^33,35^.

We compared the detected voltage from the in-house built BaSO_4_ pellet reflectance standard (S1) and an SRS-99-010 standard (S2) using the multispectral pen to assess the consistency and variability during the tissue sample measurements. The mean reflectance voltages and standard errors of the mean (SEM) were calculated across ten readings for each reflectance standard. At 850 nm, both reflectance standards showed identical mean reflectance voltages of 4.17 V with negligible distribution (SEM = 0 V). At 940 nm, S1 exhibited a mean reflectance of 5.550 V with minimal distribution (SEM = 0.00033 V), while S2 had a mean of 5.411 V with slightly higher distribution (SEM = 0.017 V). At 1050 nm, S1 demonstrated a mean reflectance of 4.170 V with a broader distribution (SEM = 0.058 V), whereas S2 had a mean of 3.242 V with a relatively narrower distribution (SEM = 0.010 V), as shown in Fig. 2B.

### Automated multispectral pen for breast tissue study

**Fig. 3:**
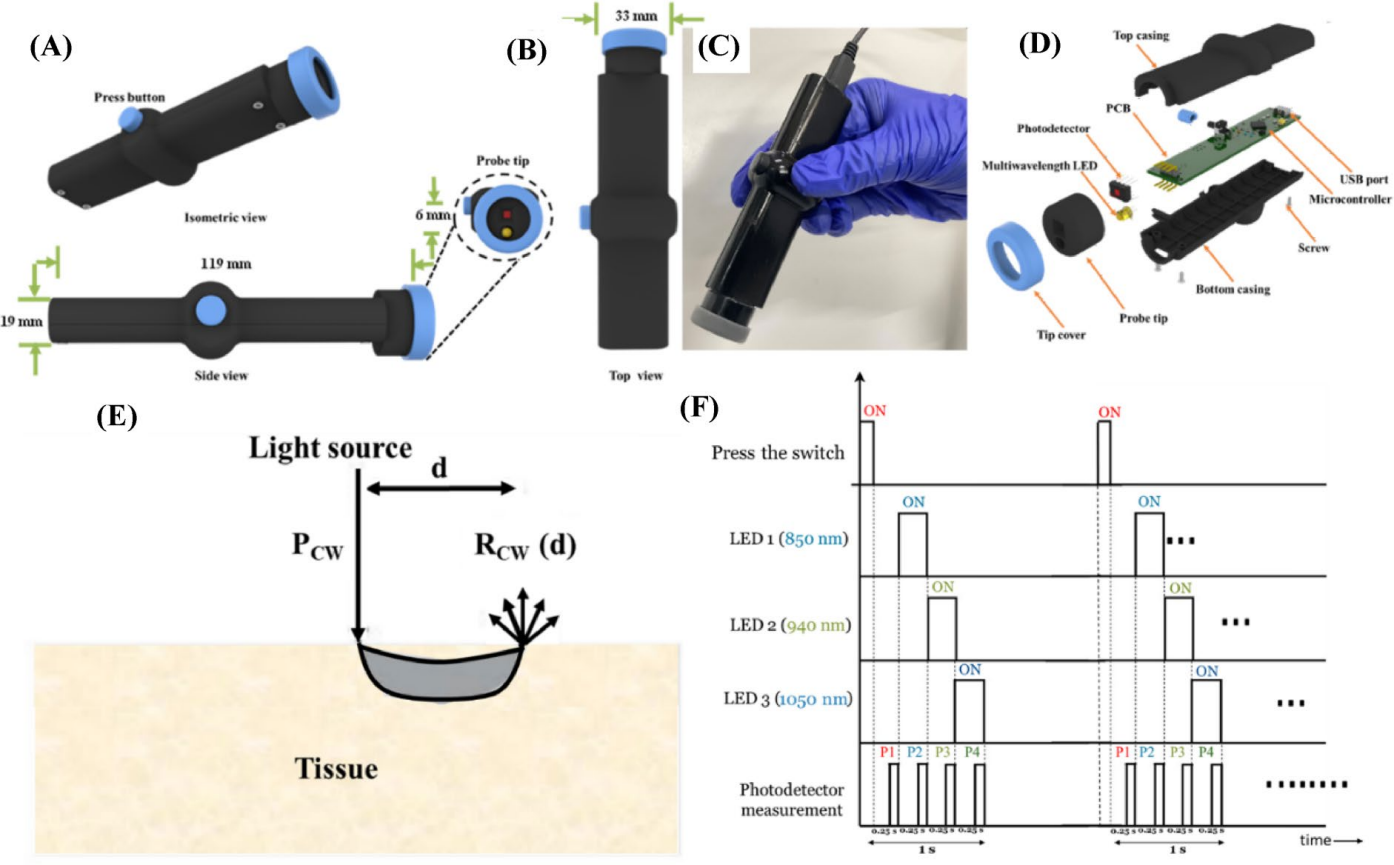
Automated DRS-based multispectral pen: (**A**) Isometric, side view and the tip (inset) of the multispectral pen, (**B**) Top view, (**C**) Actual image of the system, and (**D**) Exploded view of the automated multispectral pen, (**E**) Schematic of the working principle of the developed DRS-based Pen, and (**F**) The timing diagram of the data acquisition cycle operating at 850 nm, 940 nm, and 1050 nm, respectively.

The detachable tip of the pen has a slot for LEDs and a photodetector, which act as a light source and detector, respectively. Figure 3A and B show the different views of the multispectral pen and the detachable tip (inset). The actual developed pen held by the operator is shown in Fig. 3C. The exploded view of the multispectral pen with associated parts is shown in Fig. 3D. The engineering design of the multispectral pen is shown in Supplementary Fig. S1. The photodetector consists of a silicon photodiode integrated with an on-chip high-gain trans-impedance amplifier circuit. The automated multispectral pen’s dimensions (L x B x H) are 119 mm x 33 mm x 19 mm. All the associated parts, including the casing, were built with the help of a stereolithography (SLA) 3D printer using PLA material. The working principle of the multispectral pen integrated with a multiwavelength LED source is shown in Fig. 3E. The data acquisition is divided into four cycles of 250 ms each. The measurement process involves four distinct cycles. The first cycle detects ambient noise by activating only the photodetector. The second cycle involves measurement with the 850 nm LED switched ON. Similarly, the third cycle corresponds to measurements using the 940 nm LED, and the fourth cycle involves measurements with the 1050 nm LED, as shown in Fig. 3F. The source and detector are spaced at 6 mm, yielding a tissue probing depth of up to 3 mm or less^36^. The geometrical configuration of the source and detector, combined with the effect of optical fingerprints, also provides information regarding the probing depth^36^. To avoid significant light attenuation in the visible wavelength range, the longer wavelengths (850 nm, 940 nm, and 1050 nm) were considered, which can penetrate deep inside the tissue and interact significantly with the absorption peaks of collagen (~ 850 nm), lipids (~ 940 nm), and collagen at (~ 1050 nm)^37^. The absorption peak of the biomarkers, the limited wavelength sensitivity of the photodetector, and keeping the size and cost of the pen as minimal as possible were the main reasons behind the incorporation of only these three wavelengths.

The pen consists of a press button to begin the measurement cycle and a micro-USB-B type connector for powering and transferring the acquired data to the computer. The data was obtained and processed using a microcontroller (ATmega328P, Microchip Technology, Chandler, AZ, USA). A LabVIEW-based graphical user interface (GUI) was developed for easy interpretation of the acquired data, and a snapshot of the GUI is shown in Supplementary Fig. S1. The data processing was performed using a two-step process. In the first step, the acquired experimental data (e.g., detected voltages) is automatically stored using the GUI. In the second step, the stored data was further analyzed using a Python script to calculate the reflectance and reduced absorbance of the tissue samples. The tissue constituents (e.g., lipids and collagen) yield unique optical fingerprints (e.g., intrinsic absorption and scattering), specifically at 850 nm, 940 nm, and 1050 nm^38^. Further, the obtained reduced absorbance values were also used to classify adjacent normal and malignant breast tissue samples using an ML model.

#### Electronics module

The automated multispectral pen used a CH340G USB-UART converter that interfaced the USB with the microcontroller. A 12 MHz oscillator provided the necessary clock pulses for the CH340G. A separate 16 MHz ± 20 ppm oscillator was used to control the microcontroller. A filter and decoupling capacitor were used to obtain a regulated 5 V supply from a USB cable to drive the LED driver and photodetector circuits. A 20 V, 0.5 A MBR0520LT1G Schottky diode (ON Semiconductor, Phoenix, AZ, USA) was used between the USB and the internally regulated 6 V supply line to prevent reverse current flow to the computer from possible capacitor discharges. The LED-driver circuit consists of three TPS7A8300RGWT low-noise voltage regulators (Texas Instruments, Dallas, TX, USA) to provide the desired forward voltage to control the LED sources. The regulator outputs were designed to provide the optimum voltages for the three LEDs. The photodetector consists of a silicon-based monolithic photodiode (*OPT101*, Texas Instruments, Dallas, TX, USA) with an integrated transimpedance amplifier. The photodetector has a peak sensitivity of 850 nm, with a varying sensitivity between 750 nm and 1100 nm.

#### Optical module

Multispectral measurements were acquired using an L850/940/1050-40C00 integrated LED module (Marubeni, Tokyo, Japan) with peak emissions at 850 nm, 940 nm, and 1050 nm that correspond to collagen and lipid absorption peaks in the tissue^37,39^. Full-width half maximum (FWHM) of LEDs operating at 850 nm, 940 nm, and 1050 nm were 40 nm, 50 nm, and 80 nm, respectively, with radiant powers of 10 mW. The LED has a viewing half-angle of 50°. OPT101, which has a 14 kHz bandwidth, can detect infrared light with varied sensitivity (750 –1100 nm). The photodetector works in photoconductive mode and has a maximum quantum efficiency of 87.5% at 850 nm. The output voltage is directly proportional to the incident light intensity on the photodetector^26^.

### Ex vivo diffuse reflectance spectroscopy (DRS) measurements

The multispectral pen is operated in continuous wave (CW) mode at 850 nm, 940 nm, and 1050 nm. The relative diffuse reflectance detected by the multispectral pen was obtained by taking the ratio of the reflected light intensity from the breast tissue sample (I_tissue_) to that from an indigenously developed barium sulfate (BaSO_4_)-based reflectance standard (I_std_) as discussed by Poh et al.^34^, while accounting for ambient or dark background signals (I_bg_). Subtracting the background signal (I_bg_) from both the numerator and denominator of Eq. 1 ensures that the reflectance measurements are corrected for background interference, such as contributions from ambient light.

For a given wavelength, when measurements are taken using the photodetector under identical configurations, the reflected light intensity captured by the photodetector exhibits a linear relationship with the output voltage. Thus, the relative reflectance (R_CW_) value can be approximated in terms of detected voltage as:^40^

1 $$\:{R}_{cw}\left({\uplambda\:}\right)=\frac{{I}_{tissue}\left({\uplambda\:}\right)-{I}_{bg}\left({\uplambda\:}\right)}{{I}_{std}\left({\uplambda\:}\right)-{I}_{bg}\left({\uplambda\:}\right)}\approx\:\:\frac{{V}_{tissue}\left({\uplambda\:}\right)-{V}_{bg}\left({\uplambda\:}\right)}{{V}_{std}\left({\uplambda\:}\right)-{V}_{bg}\left({\uplambda\:}\right)}$$

Here, V_tissue_($\:{\uplambda\:}$) represents the detected voltage from the tissue sample, while V_std_($\:{\uplambda\:}$) denotes the detected voltage from the reflectance standard at a given wavelength.

According to Beer-Lambert’s law, Absorbance (A_CW_) is described as the attenuation of light intensity (I_sample_) after passing through the sample relative to the incident intensity (I_ref_) and is given as^41^:

2 $$\:{A}_{CW}=log\left(\frac{{I}_{ref\:}}{{I}_{sample}}\right)\:$$

However, in reflectance measurements, the detected intensity is influenced by both scattering and absorption processes, making it challenging to analyze these phenomena separately. To address this complexity, we can use a modified parameter called reduced absorbance (A_CWS_), defined as:^42–44^.

3 $$\:{A}_{CWS}=log\left(\frac{{I}_{s}}{{I}_{sample}}\right)$$

Where I_s_ is the light intensity measured by the detector when the concentration of absorbers are zero (i.e., only scattering is present). The attenuated light intensity (I_sample_) after passing from the tissue sample is the reflected light intensity from the tissue sample (I_tissue_) after subtracting the ambient light intensity (I_bg_). Similarly, incident intensity (I_s_) is the light intensity detected from the highly reflective reflectance standard (I_std_), such as the BaSO_4_ reflectance standard, after subtracting the background noise (I_bg_). Equation 3 is further modified as:

4 $$\:{A}_{CWS}\left({\uplambda\:}\right)=\:log\left(\frac{1}{{R}_{cw}\left({\uplambda\:}\right)}\right)=log\left(\frac{{I}_{std}\left({\uplambda\:}\right)-{I}_{bg}\left({\uplambda\:}\right)}{{I}_{tissue}\left({\uplambda\:}\right)-{I}_{bg}\left({\uplambda\:}\right)}\right)\approx\:log\left(\frac{{V}_{std}\left({\uplambda\:}\right)-{V}_{bg}\left({\uplambda\:}\right)}{{V}_{tissue}\left({\uplambda\:}\right)-{V}_{bg}\left({\uplambda\:}\right)}\right)$$

Where log is the base 10 logarithm.

The pen was calibrated before and after every data collection by measuring the background and reference light intensity. During the clinical data acquisition, the pen was placed normally over the tissue surface. Initially, only the photodetector was ON for 250 ms, and the LED was OFF, which corresponds to the background measurement (I_bg_). The diffuse reflectance measurements were acquired for *N* = 31 patients using the multispectral pen, held normally over the ex vivo tissue samples. To minimize back-reflections from the underlying surface, the tissue sample, after being removed from the container, was placed over the glass slide for 2 min so that the excess formalin could evaporate onto the glass surface. The glass slide was placed on a black background sheet while taking the measurements. Given the source-detector separation (6 mm) and the sample thickness (3 mm), a low-reflectance black background sheet improved measurement reliability by isolating the signal contribution from the surface beneath^45^. All the measurements were done using the same black background sheet. An additional experiment further demonstrated that using a different backing material (e.g., white backing) increases the reflectance measurement of tissue samples, while preserving the significant difference between cancerous and adjacent normal tissue values, irrespective of the backing used (Supplementary Fig. S4).

### Machine learning-based classification

#### Dataset

The diffuse reflectance spectroscopy (DRS) point-based measurements acquired at three specific wavelengths were used as input features to train and test these ML-based classifiers. The ground truth labels for tissue classification (adjacent normal vs. malignant) were derived from histopathological analysis (gold standard) of the tissue samples. The algorithms were evaluated on their ability to predict these histopathology-confirmed diagnoses using only the spectral absorbance data. The dataset used for model development includes reduced absorbance (A_CWS_) values at three wavelengths (850 nm, 940 nm, and 1050 nm) as input features, corresponding histopathological classifications (normal vs. malignant) as labels, and patient identifiers to group patient-specific samples.

The final classification model for our dataset was developed through a two-stage process. First, we evaluated different algorithms using nested k-fold cross-validation (as discussed in the “Algorithm selection via nested k-fold cross-validation” section) to select the best-performing algorithm. Subsequently, we performed model selection to identify the optimal hyperparameters for the chosen algorithm using k-fold cross-validation (as discussed in the “Model selection via k-fold cross-validation” section).

#### Data pre-processing

The reduced absorbance (A_CWS_) measured for each tissue sample at 850 nm, 940 nm, and 1050 nm was used as input features to the models, along with their corresponding histopathological labels and patient identifiers. Since the A_CWS_ values for all three wavelengths lie in the range of 0 and 1, so there was no need for normalization. Instead, standardization was performed using the standard scaler function from the scikit-learn library, which transformed the data to have a mean of zero and a standard deviation of one. The following formula is used for standardization:

$$\:\text{z}=\frac{x-\:\mu\:}{{\upsigma\:}}$$

Where x is the feature value, µ is the mean of the feature values, and σ is the standard deviation. This ensures that all features are on a comparable scale, which is essential for robust model performance. Outlier detection was performed using a 3σ threshold (z-score > 3) to exclude extreme values^46^. Two outliers (malignant tissue readings from samples 5 and 11 at 850 nm wavelength) were detected with z-scores of 4.33 and 3.08, respectively. As the outlier from sample 11 was only marginally above the threshold, and that from sample 5 showed deviation at a single wavelength, the complete set of measurements for both samples was retained for subsequent machine learning analysis.

#### Algorithm selection via nested k-fold cross-validation

To classify breast tissue samples as normal or malignant using reflectance values at three wavelengths, we compared seven ML algorithms: Logistic Regression, Random Forest, XGBoost, CatBoost, Support Vector Machines (SVM), k-Nearest Neighbor (kNN), and Gaussian Naive Bayes. Given the small dataset size (*n* = 50 samples), it was important to choose algorithms that are less prone to overfitting and can handle low-dimensional data effectively^32^. All ML models were developed using Python.

The primary challenge in our study was selecting the most appropriate ML algorithm and its corresponding hyperparameters for our dataset. The most common approach is the train/test split, where a portion of the data (test set) is reserved for validation before developing the model. This method also provides an unbiased performance estimate but requires holding back a significant portion of data solely for validation purposes, which is often impractical in studies involving human subjects due to the high cost and complexity of data collection.

When datasets are limited, cross-validation (CV) becomes a preferred solution. Unlike the train/test split, CV iteratively trains models on different portions of the data, allowing all data to be used for both training and validation. The k-fold CV is a popular variant, where the data is divided into k-folds. Each fold is used once as a validation set, while the remaining folds are used for training. This process is repeated k times, and the model’s performance is calculated as the average performance across all validation folds.

k-fold CV is economical with limited data, as it allows all data to be used for training and validation without needing a separate test set. It provides more accurate out-of-sample error estimates than train/test splits because it uses all available data for validation, reducing the influence of noise and making the estimates more representative of the population. However, k-fold CV does not inherently separate the data used for model development from that used for model evaluation. This can lead to over-optimistic performance estimates^47^, as pointed out by Stone^48^ and demonstrated by Varma and Simon^49^.

To address these limitations, nested k-fold cross-validation (nk-fold CV) is employed, which gives unbiased performance estimates regardless of the sample size^49–51^. nk-fold CV involves two layers of k-fold CV: an inner loop for hyperparameter tuning and model selection, and an outer loop for evaluating the selected model’s performance. This approach ensures that the data used for model evaluation is independent of the data used for model development, providing almost unbiased performance estimates. Using all available data efficiently and maintaining rigorous validation standards, nk-fold CV is particularly beneficial for small datasets where separate validation sets are not feasible.

To address the challenge of grouped data and mitigate overfitting to group-specific patterns, we employed the GroupKFold approach, a variant of k-fold cross-validation explicitly designed for datasets with inherent group structures. GroupKFold ensures that all samples within a group are exclusively assigned to either the training or validation/test set in each split, preserving group integrity and preventing data leakage. This approach provides a realistic assessment of the model’s ability to generalize to entirely unseen groups, a critical consideration in clinical and operational settings where group-specific variability is inherent.

**Fig. 4:**
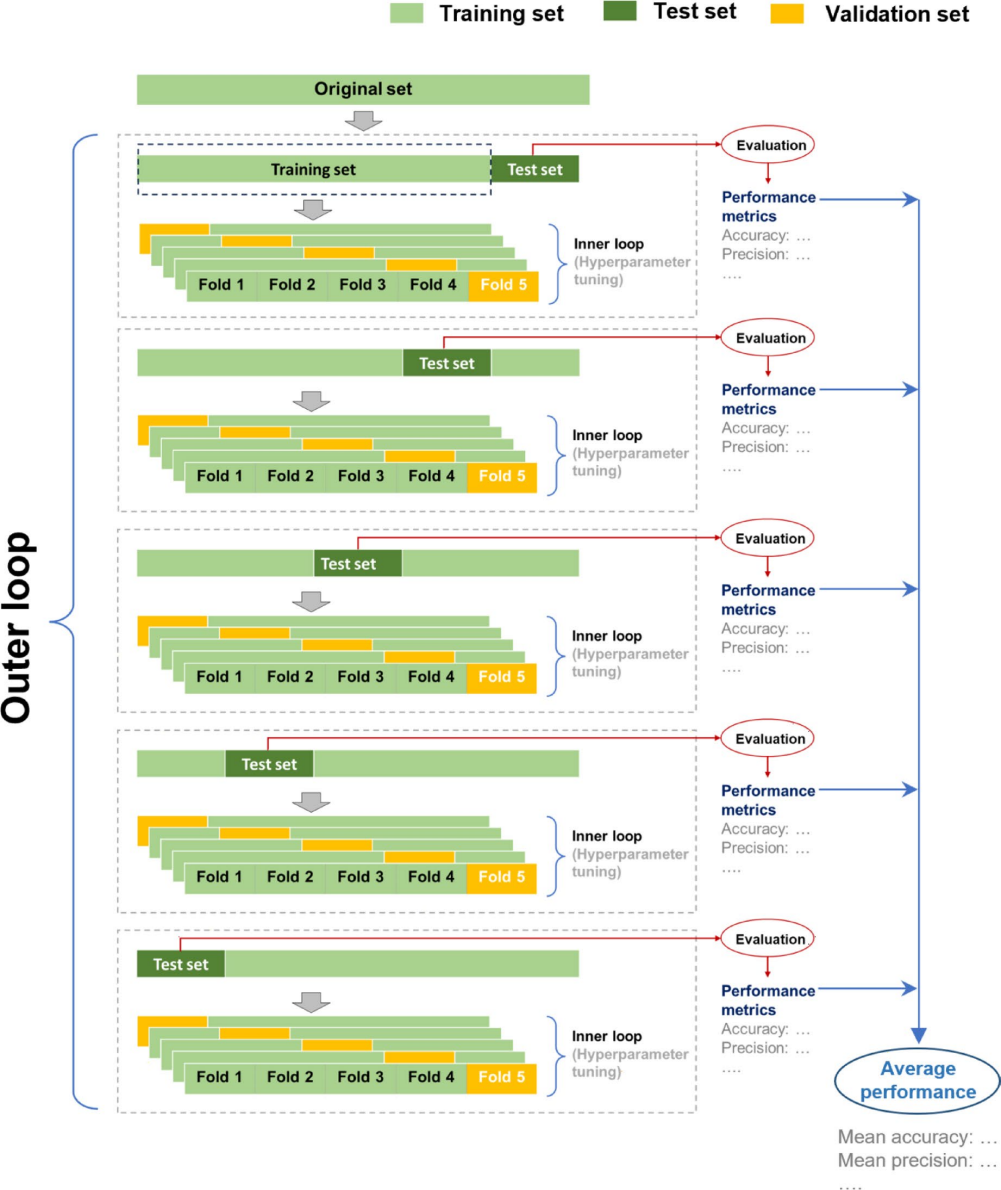
Schematic flow diagram of nested k-fold cross-validation with a 5 × 5 setup (five outer loops and five inner loops).

The nk-fold CV was implemented with a 5 × 5 setup (5-fold CV in the outer loop and 5-fold CV in the inner loop), as shown in Fig. 4. The inner loop performs model selection and hyperparameter tuning, while the outer loop estimates the model’s generalization error. Given a balanced dataset comprising *n* = 50 formalin tissue samples, twenty-five representing cancer cases and twenty-five representing normal cases, this approach aimed to maintain class proportions across all training and validation splits. In the outer loop, the dataset was partitioned into five distinct folds, each containing ten samples (five cancerous, five normal). For each outer loop iteration, forty samples (twenty cancerous, twenty normal) were used for training, while the remaining ten samples (five cancerous, five normal) served as the test set. The inner loop then further divided the training set into five folds, each comprising eight samples (four cancer, four normal), facilitating hyperparameter tuning and model selection on thirty-two training (sixteen cancerous, sixteen normal) samples and eight validation samples (four cancerous, four normal). At the same time, the balanced nature of the dataset was preserved. We employed nested k-fold cross-validation (nk-fold CV) for each algorithm to comprehensively evaluate their overall performance. This approach enabled us to select the most robust algorithm based on our specific needs, ensuring that our choice was informed by unbiased performance estimates.

**Fig. 5:**
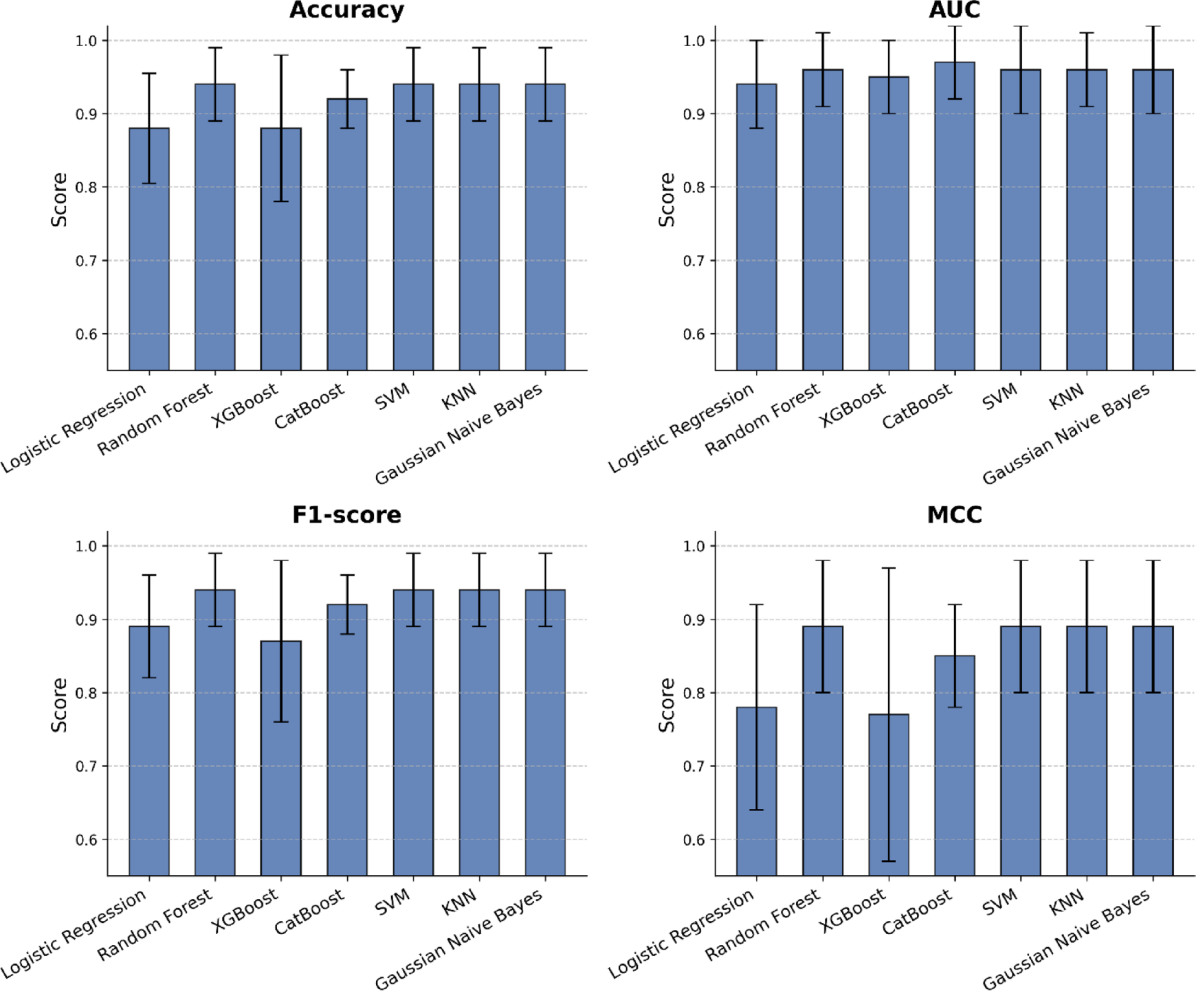
Bar plot comparing accuracy, AUC, F1 score, and MCC for all seven algorithms.

The performance metrics for each algorithm are summarized in Table 1, including accuracy, precision (PPV), recall (sensitivity), specificity, negative predictive value (NPV), F1 score, Matthews correlation coefficient (MCC), and area under the curve (AUC). The bar plot comparing accuracy, AUC, F1 score, and MCC for all seven algorithms is shown in Fig. 5. All the metrics are reported as mean ± standard deviation. Supplementary Table 2 contains all the hyperparameters and their range used for tuning the models.

**Table 1:** Performance metrics obtained using nested k-fold cross-validation for all classification algorithms.

Model	Accuracy	Precision (PPV)	Sensitivity	Specificity	NPV	F1 Score	MCC	AUC
Logistic Regression	0.88 ± 0.075	0.87 ± 0.11	0.92 ± 0.10	0.84 ± 0.15	0.93 ± 0.09	0.89 ± 0.07	0.78 ± 0.14	0.94 ± 0.06
Random Forest	0.94 ± 0.05	0.93 ± 0.08	0.96 ± 0.08	0.92 ± 0.10	0.97 ± 0.07	0.94 ± 0.05	0.89 ± 0.09	0.96 ± 0.05
XGBoost	0.88 ± 0.10	0.88 ± 0.10	0.88 ± 0.16	0.88 ± 0.10	0.90 ± 0.13	0.87 ± 0.11	0.77 ± 0.20	0.95 ± 0.05
CatBoost	0.92 ± 0.04	0.93 ± 0.08	0.92 ± 0.10	0.92 ± 0.10	0.93 ± 0.08	0.92 ± 0.04	0.85 ± 0.07	0.97 ± 0.05
SVM	0.94 ± 0.05	0.97 ± 0.07	0.92 ± 0.10	0.96 ± 0.08	0.93 ± 0.08	0.94 ± 0.05	0.89 ± 0.09	0.96 ± 0.06
KNN	0.94 ± 0.05	0.97 ± 0.07	0.92 ± 0.10	0.96 ± 0.08	0.93 ± 0.08	0.94 ± 0.05	0.89 ± 0.09	0.96 ± 0.05
Gaussian Naive Bayes	0.94 ± 0.05	0.93 ± 0.08	0.96 ± 0.08	0.92 ± 0.10	0.97 ± 0.07	0.94 ± 0.05	0.89 ± 0.09	0.96 ± 0.06

CatBoost was chosen as the final model to perform classification due to its good balance between discriminative power, robustness, and generalizability. CatBoost achieved the highest AUC-ROC among all models, indicating an exceptional ability to distinguish between positive and negative classes. Unlike other models that exhibited a trade-off between sensitivity and specificity (e.g., Random Forest with higher sensitivity but slightly lower specificity), CatBoost maintained a balance between correctly identifying true positives and avoiding false positives. CatBoost exhibited lower standard deviations in accuracy (± 0.04) and MCC (± 0.07) compared to alternatives like XGBoost (± 0.10 in accuracy, ± 0.20 in MCC), suggesting greater consistency across different validation sets. CatBoost achieved a Matthews Correlation Coefficient (MCC) score of 0.85, reflecting a good overall correlation between predicted and actual outcomes by considering all components of the confusion matrix. Although slightly lower than the MCCs of Random Forest, SVM, KNN, and Gaussian Naive Bayes model (≈ 0.89), CatBoost’s higher AUC and well-balanced sensitivity and specificity provide more reliable and clinically relevant performance.

While the SVM and KNN models showed slightly higher accuracy (0.94) and precision (0.97), this came with a modest reduction in sensitivity (0.92 compared to 0.96 in Random Forest and Gaussian Naive Bayes). In contrast, the CatBoost model performs well across all key metrics, making it a more practical and adaptable choice for clinical applications.

#### Model selection via k-fold cross-validation

**Fig. 6:**
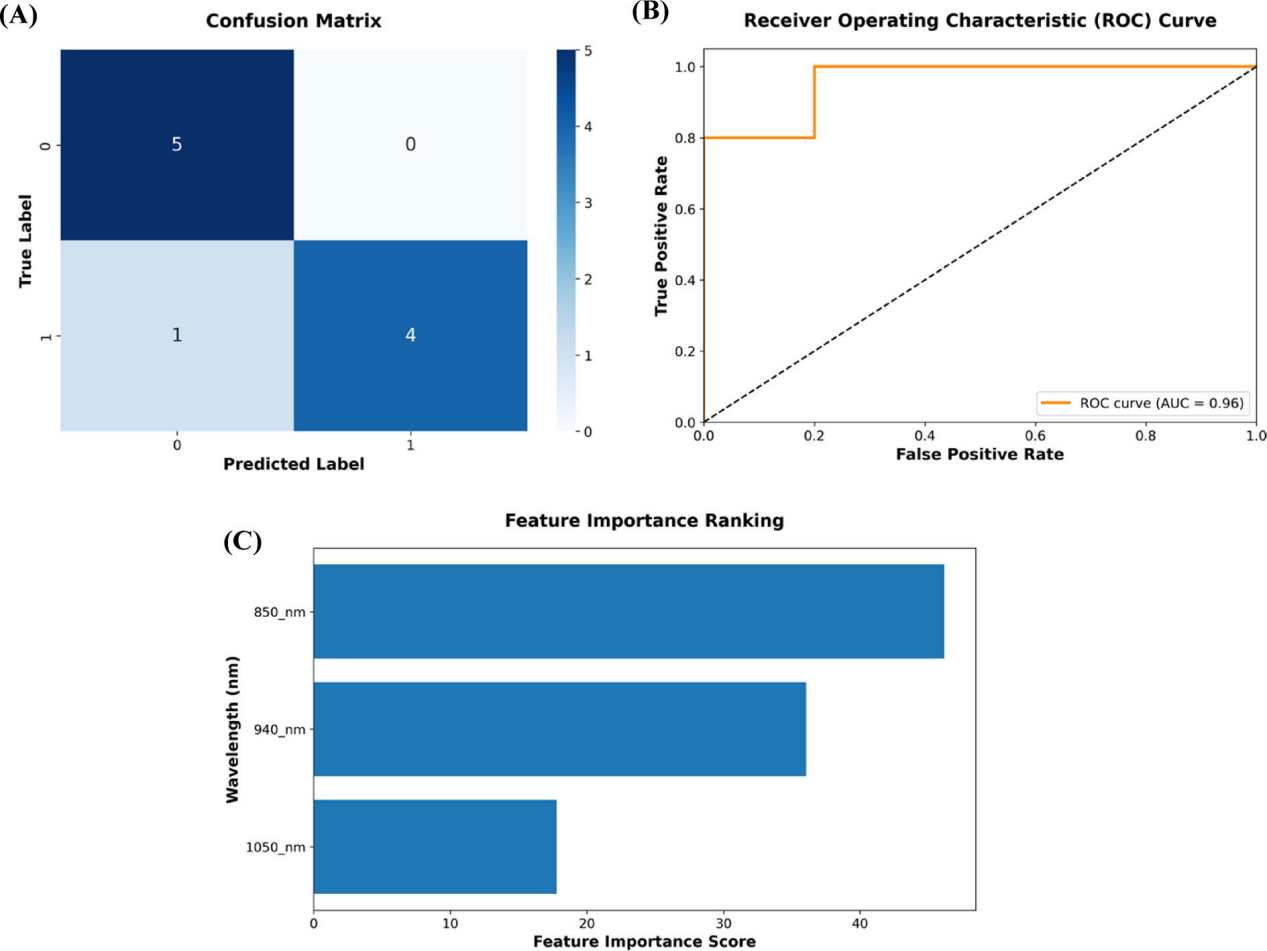
Final CatBoost model: (**A**) Confusion matrix, (**B**) ROC curve, and (**C**) Feature importance score.

After identifying the best algorithm suited for our application, i.e., CatBoost, the next step is to perform model selection (i.e., hyperparameter tuning) via cross-validation. A stratified k-Fold cross-validation strategy (5-fold) was adopted to preserve label distribution while avoiding data leakage across groups during model training and evaluation. For hyperparameter optimization, we utilized RandomizedSearchCV with 50 iterations, searching across a defined space for key CatBoost parameters: number of iterations, tree depth, learning rate, L2 regularization, and border count. The optimal model, selected based on the best cross-validation performance, was subsequently retrained on the entire training set using the best hyperparameters obtained. Performance evaluation of the final CatBoost model was conducted on a held-out test set, which maintained group-level stratification. Key metrics—including accuracy, precision (PPV), recall (sensitivity), specificity, negative predictive value (NPV), F1 score, Matthews correlation coefficient (MCC), and area under the curve (AUC) were computed to assess classification efficacy, as described in Table 2. Additionally, an analysis of feature importance was conducted to interpret the contribution of individual wavelengths to classification performance. The confusion matrix, ROC curve, and feature importance score of the finalized CatBoost model are shown in Fig. 6.

**Table 2:** Performance metrics of the final catboost model.

Model	Accuracy	Precision (PPV)	Sensitivity	Specificity	NPV	F1 Score	MCC	AUC
CatBoost	0.9000	1.00	0.80	1.00	0.83	0.88	0.82	0.96

### Statistical analysis

The Mann-Whitney U test, a non-parametric statistical test, was employed to assess differences between groups. This test is robust to outliers as it relies on rank-based comparisons rather than absolute values. Given the small sample size, the Mann-Whitney U test was chosen for its interpretability and suitability for non-normally distributed data. All statistical analyses were conducted using OriginPro (2024b, OriginLab Corporation, Northampton, MA, USA).

#### Ethics approval and consent to participate

The institutional ethics committee of the Indian Institute of Science and Assam Medical College approved the methods and all experimental protocols. All methods were performed per relevant guidelines and regulations per the protocol approved by the institutional ethics committee of the Indian Institute of Science and Assam Medical College. The patient’s written informed consent was obtained from all the subjects per the protocol submitted to the institutional ethics committee of the Indian Institute of Science and Assam Medical College, bearing ethical clearance certificate numbers 17-14012020 and AMC/EC/311.

## Results

### Malignant tissue samples have significantly higher diffuse reflectance and lower reduced absorbance than adjacent normal tissue

The experiments were performed using ex vivo breast tissue samples: *n* = 50 formalin-fixed tissues and *n* = 12 fresh tissue samples obtained from *N* = 25 and *N* = 6 breast cancer patients, respectively.

### Formalin-fixed tissue experiments (*N* = 25 patients)

**Fig. 7:**
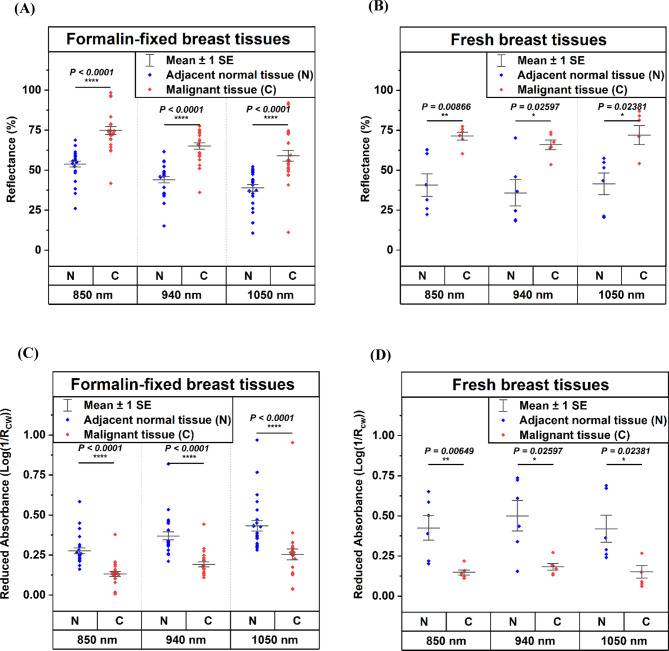
Results obtained using multispectral pen on excised tissue samples: Scatter interval plots of reflectance (%) for (**A**) Formalin-fixed tissue samples (*N* = 25 patients) and (**B**) Fresh tissue samples (*N* = 6 patients) at wavelengths of 850 nm, 940 nm, and 1050 nm, respectively. Scatter interval plots of reduced absorbance for (**C**) Formalin-fixed tissue samples (*N* = 25 patients) and (**D**) Fresh tissue samples (*N* = 6 patients) at wavelengths of 850 nm, 940 nm, and 1050 nm, respectively.

The mean reflectance < R_CW_> representing the measure of backscattered light from the tissue samples was measured three times in reflectance mode configuration. The < R_CW_> +/- SEM with *N* = 25 breast cancer patients were higher than the adjacent normal breast tissue (0.75 ± 0.02 vs. 0.54 ± 0.02 at 850 nm, 0.65 ± 0.02 vs. 0.44 ± 0.02 at 940 nm, and 0.59 ± 0.03 vs. 0.39 ± 0.02 at 1050 nm), each difference is highly statistically significant (*p* < 0.0001), as shown in Table 3; Fig. 7A, respectively. For *N* = 25, the corresponding 95% confidence intervals (CI) are: 0.75 (0.71–0.79) vs. 0.54 (0.50–0.58) at 850 nm, 0.65 (0.61–0.69) vs. 0.44 (0.40–0.48) at 940 nm, and 0.59 (0.53–0.65) vs. 0.39 (0.35–0.43) at 1050 nm. The mean reduced absorbance values <$\:{\text{A}}_{\text{C}\text{W}\text{S}}$> +/- SEM with *N* = 25 breast cancer patients was lower than the adjacent normal breast tissue (0.13 ± 0.02 vs. 0.28 ± 0.02 at 850 nm, 0.19 ± 0.01 vs. 0.37 ± 0.02 at 940 nm, and 0.25 ± 0.03 vs. 0.43 ± 0.03 at 1050 nm), each difference is highly statistically significant (*p* < 0.0001), as shown in Table 3; Fig. 7C, respectively. The corresponding confidence intervals (CI) for *N* = 25 are: 0.13 (0.09–0.17) vs. 0.28 (0.24–0.32) at 850 nm, 0.19 (0.15–0.23) vs. 0.37 (0.33–0.41) at 940 nm, and 0.25 (0.19–0.31) vs. 0.43 (0.37–0.49) at 1050 nm.

**Table 3:** **R**eflectance (%) and reduced absorbance values for formalin-fixed tissue samples (*N* = 25 patients) at 850 nm, 940 nm, and 1050 nm, respectively.

Patient No.	Reflectance (Rcw)						Reduced Absorbance (A_CWS_)					
	850 nm		940 nm		1050 nm		850 nm		940 nm		1050 nm	
	*N*	C	*N*	C	*N*	C	*N*	C	*N*	C	*N*	C
1	0.43	0.75	0.34	0.69	0.41	0.67	0.37	0.13	0.47	0.16	0.39	0.18
2	0.38	0.66	0.35	0.75	0.41	0.56	0.42	0.18	0.45	0.12	0.39	0.25
3	0.50	0.63	0.47	0.53	0.52	0.53	0.31	0.20	0.33	0.28	0.28	0.27
4	0.57	0.64	0.49	0.57	0.48	0.56	0.24	0.19	0.31	0.25	0.32	0.25
5	0.36	0.62	0.29	0.72	0.35	0.73	0.45	0.21	0.53	0.14	0.45	0.14
6	0.57	0.75	0.46	0.74	0.41	0.67	0.25	0.13	0.34	0.13	0.39	0.17
7	0.48	0.79	0.36	0.75	0.34	0.74	0.32	0.10	0.45	0.13	0.47	0.13
8	0.54	0.72	0.39	0.51	0.37	0.59	0.27	0.14	0.41	0.29	0.43	0.23
9	0.56	0.70	0.46	0.65	0.49	0.54	0.25	0.16	0.34	0.19	0.31	0.27
10	0.55	0.83	0.48	0.75	0.44	0.73	0.26	0.08	0.32	0.13	0.36	0.14
11	0.56	0.72	0.46	0.73	0.41	0.74	0.25	0.14	0.34	0.14	0.39	0.13
12	0.56	0.74	0.49	0.61	0.43	0.52	0.25	0.13	0.31	0.22	0.37	0.29
13	0.59	0.75	0.47	0.68	0.37	0.47	0.23	0.13	0.33	0.17	0.43	0.33
14	0.6	0.74	0.52	0.60	0.43	0.54	0.22	0.13	0.28	0.22	0.37	0.27
15	0.57	0.72	0.48	0.66	0.5	0.51	0.25	0.15	0.32	0.18	0.31	0.29
16	0.59	0.69	0.46	0.59	0.44	0.49	0.23	0.16	0.34	0.23	0.35	0.31
17	0.56	0.73	0.49	0.61	0.51	0.41	0.25	0.14	0.31	0.21	0.30	0.39
18	0.58	0.75	0.56	0.59	0.37	0.50	0.23	0.13	0.25	0.23	0.43	0.30
19	0.61	0.73	0.55	0.59	0.50	0.51	0.21	0.14	0.26	0.23	0.30	0.29
20	0.26	0.42	0.15	0.36	0.11	0.11	0.58	0.38	0.82	0.44	0.97	0.95
21	0.69	0.96	0.62	0.75	0.49	0.75	0.16	0.02	0.21	0.13	0.31	0.13
22	0.57	0.98	0.35	0.78	0.17	0.92	0.25	0.01	0.45	0.11	0.77	0.04
23	0.60	0.97	0.35	0.73	0.26	0.57	0.22	0.02	0.46	0.14	0.58	0.25
24	0.65	0.79	0.47	0.59	0.29	0.47	0.19	0.11	0.33	0.23	0.53	0.33
25	0.52	0.96	0.49	0.75	0.24	0.91	0.28	0.02	0.31	0.13	0.63	0.04
Mean	0.54	0.75	0.44	0.65	0.39	0.59	0.28	0.13	0.37	0.19	0.43	0.25
SEM	0.02	0.02	0.02	0.02	0.02	0.03	0.02	0.02	0.02	0.02	0.03	0.03
95% CI	0.50–0.58	0.71–0.79	0.40–0.48	0.61–0.69	0.35–0.43	0.53–0.65	0.24–0.32	0.09–0.17	0.33–0.41	0.15–0.23	0.37–0.49	0.19–0.31

### Fresh tissue experiments (*N* = 6 patients)

During the experiments with fresh breast tissue samples, the mean reflectance value < R_CW_> +/- SEM with six breast cancer patients were higher than the adjacent normal breast tissue (0.71 ± 0.01 vs. 0.41 ± 0.03 at 850 nm *p* = 8.66e-03, 0.66 ± 0.01 vs. 0.36 ± 0.04 at 940 nm *p* = 2.60e-02, and 0.72 ± 0.03 vs. 0.42 ± 0.03 at 1050 nm *p* = 2.38e-02) as shown in Table 4; Fig. 7B, respectively. For *N* = 6, the corresponding 95% confidence intervals (CI) are: 0.71 (0.63–0.79) vs. 0.41 (0.23–0.59) at 850 nm, 0.66 (0.58–0.74) vs. 0.36 (0.15–0.57) at 940 nm, and 0.72 (0.57–0.87) vs. 0.42 (0.24–0.60) at 1050 nm. The mean reduced absorbance < A_CWS_> +/- SEM with six breast cancer patients was lower than the adjacent normal breast tissue (0.15 ± 0.01 vs. 0.43 ± 0.04 at 850 nm *p* = 6.49e-03, 0.18 ± 0.01 vs. 0.50 ± 0.05 at 940 nm *p* = 2.60e-2, and 0.15 ± 0.02 vs. 0.42 ± 0.04 at 1050 nm *p* = 2.38e-02), as shown in Table 4; Fig. 7D, respectively. The corresponding confidence intervals (CI) for *N* = 6 are: 0.15 (0.10–0.20) vs. 0.43 (0.22–0.64) at 850 nm, 0.18 (0.13–0.23) vs. 0.50 (0.27–0.73) at 940 nm, and 0.15 (0.05–0.25) vs. 0.42 (0.21–0.63) at 1050 nm. The lower statistical significance for higher wavelengths may be because of lower scattering with the heterogeneous tissues compared to shorter-wavelength light^30,52^. The raw detected voltages obtained from the formalin-fixed and fresh tissues are mentioned in the Supplementary Tables S3, S4, and Fig. S2, respectively.

**Table 4:** Reflectance (%) and reduced absorbance values for fresh tissue samples (*N* = 6 patients) at 850 nm, 940 nm, and 1050 nm, respectively.

Patient No.	Reflectance (*R*_CW_)						Reduced Absorbance (A_CWS_)					
	850 nm		940 nm		1050 nm		850 nm		940 nm		1050 nm	
	*N*	C	*N*	C	*N*	C	*N*	C	*N*	C	*N*	C
1	0.63	0.60	0.46	0.54	0.21	0.54	0.20	0.22	0.34	0.27	0.67	0.27
2	0.41	0.74	0.37	0.64	0.57	0.81	0.39	0.13	0.44	0.19	0.24	0.09
3	0.6	0.69	0.7	0.74	0.43	0.54	0.22	0.16	0.15	0.13	0.36	0.27
4	0.32	0.77	0.25	0.65	0.55	0.84	0.50	0.11	0.61	0.19	0.26	0.08
5	0.26	0.72	0.18	0.72	0.52	0.71	0.59	0.14	0.74	0.14	0.29	0.15
6	0.22	0.76	0.19	0.68	0.21	0.87	0.65	0.12	0.72	0.17	0.69	0.06
Mean	0.41	0.71	0.36	0.66	0.42	0.72	0.43	0.15	0.50	0.18	0.42	0.15
SEM	0.07	0.03	0.08	0.03	0.07	0.06	0.08	0.02	0.09	0.02	0.08	0.04
95% CI	0.23–0.59	0.63–0.79	0.15–0.57	0.58–0.74	0.24–0.60	0.57–0.87	0.22–0.64	0.10–0.20	0.27–0.73	0.13–0.23	0.21–0.63	0.05–0.25

## Discussion

We have shown that our multispectral pen can reliably distinguish malignant and benign breast tissue in tissue samples, at high levels of sensitivity and specificity. These results are highly encouraging. The experiments were performed using ex vivo breast tissue samples: *n* = 50 formalin-fixed tissues and *n* = 12 fresh tissue samples were obtained from *N* = 25 and *N* = 6 breast cancer patients, respectively. Each breast tissue sample was scanned using the multispectral pen. The infiltration and growth of cancerous cells cause the malignant tissues to be heterogeneous, resulting in numerous discontinuities and gradients in the refractive indices^20,53,54^. In malignant breast tissue, the effects mentioned above lead to a higher density of the scattering microstructures, which increases the scattering compared to adjacent normal breast tissue^25^. Diffuse reflectance measurement and an ML-based classification model are adopted to quantify the bulk optical properties of the tissue samples using reflectance values obtained while operating at 850 nm, 940 nm, and 1050 nm. The process flow for selecting an ML model and algorithm was shown in Supplementary Fig. S3. The DRS technique operates on the premise that light is delivered to the tissue, and the back-scattered light is measured using a detector adjacent to the light source. The reflected light will contain information about absorption (i.e., biomolecular content) and scattering (i.e., microstructures). The spacing between the light source and detector, and the combination of scattering and absorption properties, describe the light propagation depth inside the tissue^55,56^. The biomolecular constituents of the excised tissue samples are mainly composed of lipids and collagen^57^. The present study shows that the malignant tissue constitutes more scattering elements than adjacent normal tissues due to abnormal cell growth, leading to more heterogeneity and thus resulting in more scattering of light. This observation confirms the findings of the earlier studies^58,59^. In contrast, the tissue microstructure is influenced by many factors, such as the number of mitochondria, extracellular matrix, refractive index mismatch due to vessels and fibers, size of the cells, cell membranes, collagen fibers, and other factors^60^. The effect of formalin fixation on malignant tissue is minimal and can be ignored^61,62^. The microarchitecture of the extracellular space and the intracellular spacing considerably vary by the processes of carcinogenesis and cancer progression^63,64^.

The multispectral pen is designed keeping in mind the anticipated potential for in vivo use, which necessitates a compact form factor, isolation from background noise, biocompatibility, and seamless integration with existing surgical tools. To achieve a pen-sized probe, specific wavelengths were selected. LEDs were chosen over halogen lamps or bulky lasers for illumination due to their significantly smaller size, although this decision precluded the use of a continuous spectrum of wavelengths. LEDs also offer power efficiency and non-ionizing radiation, paving the way for future battery-powered operation, unlike the wired probe configuration presented in this study. A limitation of LEDs is their lack of coherence (i.e., partially coherent source). Additionally, the relatively large source-detector distance (SDD) may cause the detector to capture light from deeper tissue regions, further reducing the intensity of the detected light and potentially decreasing overall resolution. Another characteristic of LEDs is their spectral bandwidth, which can result in frequency spreading. The electronic module is currently encapsulated within the probe body, potentially inducing electronic noise during in vivo testing. A redesigned configuration to mitigate this issue is under development. The present study focuses primarily on the effects of tissue heterogeneity on optical parameters and does not address dependencies related to applied pressure during measurements. This study is constrained by few limitations:

1. This study is a pilot study performed on a limited number of tissue samples, as the dataset comprised only 31 patients, which may not be representative of broader populations.
2. The developed device needs different clinical and regulatory approvals before conducting an in vivo animal model study, followed by human clinical trials. Afterwards, this technology can only go for full incorporation into the clinical workflow.
3. The explanation of the ML model employed in the study could be expanded, especially regarding its ability to handle categorical data.
4. This study lacks the validation of the developed multispectral pen on in vivo patient samples, as a limited number of formalin-fixed and fresh tissue samples were analyzed.

Based on the results obtained using limited formalin-fixed and fresh tissue samples, we envisage performing similar studies on a large cohort of ex vivo samples followed by an in vivo animal model study. The high accuracy found in this study will help us get the necessary ethics and IRB approvals for future in vivo studies in animals and patients.

## Conclusion

In this study, we designed and developed a compact, low-cost multispectral pen intended to assist clinicians in the rapid identification of breast cancer and differentiate benign and malignant breast tissue. The device demonstrated excellent ex vivo performance, successfully delineating adjacent normal and malignant formalin-fixed tissue samples based on reduced absorbance values. Using the optimized CatBoost model, we achieved a sensitivity of 80%, specificity of 100%, and overall accuracy of 90%. The model also yielded a Matthews Correlation Coefficient (MCC) of 0.82, a positive predictive value (PPV) of 100%, and a negative predictive value (NPV) of 83%, indicating its strong discriminative capability.

These results highlight the potential of the multispectral pen as an effective diagnostic aid for distinguishing malignant from adjacent normal breast tissues with high statistical confidence in ex vivo settings. These results should prompt testing the accuracy of this device to assess breast biopsy specimens in a larger patient population. Building on this foundation, we are currently developing a multimodal intraoperative probe designed for real-time, in vivo applications. Future work will focus on validating the device after integrating with machine learning techniques on a larger cohort of clinical samples to further assess its robustness, generalizability, and clinical utility across diverse tissue types and patient populations.

## Supplementary Information

Below is the link to the electronic supplementary material.

Supplementary Material 1

## Data Availability

The datasets used or analyzed during the current study are available from the corresponding author upon reasonable request.
