# Supplementary material for: Design and development of a portable multiwavelength LED-based diffuse reflectance spectroscopy tool for rapid breast cancer identification
Source: Sci Rep. 2025 Nov 25;15:41859. doi: 10.1038/s41598-025-25867-8 (PMC12647633; doi:10.1038/s41598-025-25867-8)

***Supplementary Information***

***For***

Design and development of a portable multiwavelength LED-based diffuse reflectance spectroscopy tool for rapid breast cancer identification

Arif Mohd. Kamal^a^, Kirteyman Singh Rajput^a,ǂ^, Apurva Dahake^a,ǂ^, Gayatri Gogoi^b^, Ajay Krishnan Ajith^a^, VSN Sitaramgupta Vangeti^a^, Dilip Kiling^c^, Jayant S. Vaidya^d^, and Hardik J. Pandya^a,e,^*

^a^Department of Electronic Systems Engineering, Indian Institute of Science, Bangalore, India; ^b^Medical Research Unit Laboratory and Department of Pathology, Assam Medical College, Dibrugarh, India;  ^c^Department of Surgery, Assam Medical College, Dibrugarh, India; ^d^Division of Surgery and Interventional Science, University College London, London, UK; ^e^Department of Design and Manufacturing, Indian Institute of Science, Bangalore, India.

*Correspondence: Hardik J. Pandya ([hjpandya@iisc.ac.in](mailto:hjpandya@iisc.ac.in))

^ǂ^Equal Contribution

**Tables**:

Table S1: Clinical diagnosis and Histopathological impressions of the human breast tissue samples

| **Sample No.** | **Clinical Diagnosis** | | | | **Histopathology**  **Impression** |
| --- | --- | --- | --- | --- | --- |
|  | **Menopause** | **Type of Breast Cancer** | **Age (Yrs.)** | **BMI (Kg/m^2^)** |  |
| 1 | Post | Invasive lobular Carcinoma | 56 | 23.4 | 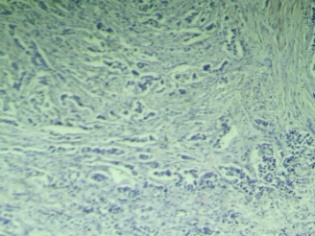  **Malignant** |
|  |  |  |  |  | 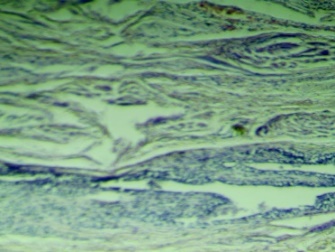  **Adjacent Normal** |
| 2 | Post | Invasive Ductal Carcinoma | 50 | 19.9 | 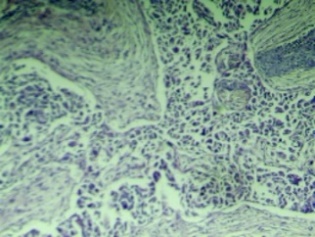  **Malignant** |
|  |  |  |  |  | 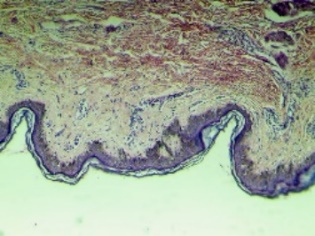  **Adjacent Normal** |

Table S2: Hyperparameters used in grid search during model tuning

| **Algorithm** | **Hyperparameter** | **Range** |
| --- | --- | --- |
| Logistic Regression | classifier__C | 0.001, 0.01, 0.1, 1, 10, 100 |
|  | classifier__penalty | 'l1', 'l2', 'elasticnet', None |
|  | classifier__solver | 'newton-cg', 'lbfgs', 'liblinear', 'sag', 'saga' |
|  | classifier__max_iter | 50, 100, 200, 500 |
| Random Forest | classifier__n_estimators | 100, 200, 300, 400, 500 |
|  | classifier__max_depth | 3, 4, 5, 6, None |
|  | classifier__min_samples_split | 2, 5, 10 |
|  | classifier__min_samples_leaf | 1, 2, 4 |
|  | classifier__max_features | 'sqrt', 'log2' |
|  | classifier__bootstrap | True, False |
| XGBoost | classifier__n_estimators | 50, 100, 200 |
|  | classifier__max_depth | 3, 5, 7 |
|  | classifier__learning_rate | 0.01, 0.1, 0.2 |
|  | classifier__subsample | 0.6, 0.8, 1.0 |
|  | classifier__colsample_bytree | 0.6, 0.8, 1.0 |
|  | classifier__gamma | 0, 0.1, 0.2 |
|  | classifier__reg_alpha | 0, 0.1, 1 |
|  | classifier__reg_lambda | 0, 0.1, 1 |
| CatBoost | classifier_iterations | 100, 200, 300 |
|  | classifier_depth | 4, 6, 8 |
|  | classifier__learning_rate | 0.01, 0.05, 0.1 |
|  | classifier__l2_leaf_reg | 1, 3, 5 |
|  | classifier__border_count | 32, 64, 128 |
|  | classifier__random_strength | 0.5, 1, 2 |
|  | classifier__bagging_temperature | 0, 0.5, 1 |
|  | classifier__verbose | False |
| SVM | classifier__C | 0.1, 1, 10, 100 |
|  | classifier__gamma | 'scale', 'auto', 0.01, 0.1, 1 |
|  | classifier__kernel | 'linear', 'rbf', 'poly' |
|  | classifier__degree | 2, 3, 4 |
|  | classifier__probability | True |
| KNN | classifier__n_neighbors | 3, 5, 7, 9, 11, 13 |
|  | classifier__weights | 'uniform', 'distance' |
|  | classifier__p | 1, 2 |
|  | classifier__metric | 'minkowski', 'cosine' |
| Gaussian Naive Bayes | classifier__var_smoothing | 1e-9, 1e-8, 1e-7, 1e-6, 1e-5 |

Table S3: Detected voltage values for formalin-fixed tissue samples (N=25 patients) at 850 nm, 940 nm, and 1050 nm.

|  | **Detected Voltage (V_d_)** | | | | | |
| --- | --- | --- | --- | --- | --- | --- |
| **Patient No.** | **850 nm** | | **940 nm** | | **1050 nm** | |
|  | **N** | **C** | **N** | **C** | **N** | **C** |
| 1 | 1.62 | 2.81 | 0.86 | 1.74 | 0.69 | 1.12 |
| 2 | 1.45 | 2.49 | 0.89 | 1.90 | 0.69 | 0.94 |
| 3 | 1.87 | 2.36 | 1.19 | 1.34 | 0.88 | 0.89 |
| 4 | 2.15 | 2.43 | 1.24 | 1.43 | 0.80 | 0.94 |
| 5 | 1.34 | 2.34 | 0.74 | 1.81 | 0.59 | 1.23 |
| 6 | 2.14 | 2.82 | 1.16 | 1.86 | 0.69 | 1.12 |
| 7 | 1.83 | 2.97 | 0.90 | 1.89 | 0.57 | 1.24 |
| 8 | 2.04 | 2.73 | 0.99 | 1.29 | 0.62 | 0.99 |
| 9 | 2.12 | 2.62 | 1.17 | 1.65 | 0.81 | 0.91 |
| 10 | 2.08 | 3.14 | 1.21 | 1.89 | 0.74 | 1.22 |
| 11 | 2.10 | 2.72 | 1.15 | 1.84 | 0.69 | 1.25 |
| 12 | 2.11 | 2.79 | 1.24 | 1.53 | 0.72 | 0.87 |
| 13 | 2.21 | 2.82 | 1.18 | 1.72 | 0.63 | 0.78 |
| 14 | 2.27 | 2.78 | 1.32 | 1.52 | 0.71 | 0.91 |
| 15 | 2.14 | 2.70 | 1.22 | 1.67 | 0.83 | 0.86 |
| 16 | 2.23 | 2.61 | 1.16 | 1.48 | 0.74 | 0.83 |
| 17 | 2.11 | 2.74 | 1.24 | 1.54 | 0.85 | 0.69 |
| 18 | 2.21 | 2.81 | 1.41 | 1.49 | 0.62 | 0.84 |
| 19 | 2.32 | 2.77 | 1.39 | 1.48 | 0.84 | 0.86 |
| 20 | 0.98 | 1.58 | 0.38 | 0.91 | 0.18 | 0.19 |
| 21 | 2.6 | 3.62 | 1.55 | 1.89 | 0.82 | 1.25 |
| 22 | 2.14 | 3.71 | 0.89 | 1.96 | 0.29 | 1.54 |
| 23 | 2.28 | 3.65 | 0.87 | 1.84 | 0.44 | 0.95 |
| 24 | 2.47 | 2.96 | 1.17 | 1.49 | 0.49 | 0.79 |
| 25 | 1.97 | 3.63 | 1.24 | 1.89 | 0.40 | 1.53 |
| **Mean** | **2.03** | **2.82** | **1.11** | **1.64** | **0.65** | **0.99** |
| **SEM** | **0.07** | **0.10** | **0.05** | **0.05** | **0.04** | **0.06** |

Table S4: Detected voltage values for fresh tissue samples (N=6 patients) at 850 nm, 940 nm, and 1050 nm.

|  | **Detected Voltage (V_D_)** | | | | | |
| --- | --- | --- | --- | --- | --- | --- |
| **Patient No.** | **850 nm** | | **940 nm** | | **1050 nm** | |
|  | **N** | **C** | **N** | **C** | **N** | **C** |
| 1 | 2.37 | 2.28 | 1.16 | 1.35 | 0.36 | 0.91 |
| 2 | 1.54 | 2.79 | 0.93 | 1.61 | 0.96 | 1.36 |
| 3 | 2.28 | 2.62 | 1.77 | 1.86 | 0.73 | 0.91 |
| 4 | 1.19 | 2.92 | 0.62 | 1.63 | 0.92 | 1.41 |
| 5 | 0.98 | 2.71 | 0.46 | 1.83 | 0.86 | 1.19 |
| 6 | 0.84 | 2.85 | 0.48 | 1.70 | 0.34 | 1.46 |
| **Mean** | **1.53** | **2.7** | **0.9** | **1.66** | **0.7** | **1.21** |
| **SEM** | **0.27** | **0.09** | **0.26** | **0.08** | **0.11** | **0.10** |

**Figures:**

**Fig. S1:** Engineering design of Multispectral Pen and GUI. (a) Exterior view of the Pen, (b) Interior view of the Pen, (c) Different views of the Pen’s tip, (d)Associated parts of the Pen, and (e) Snapshot of the developed LabVIEW-based GUI.


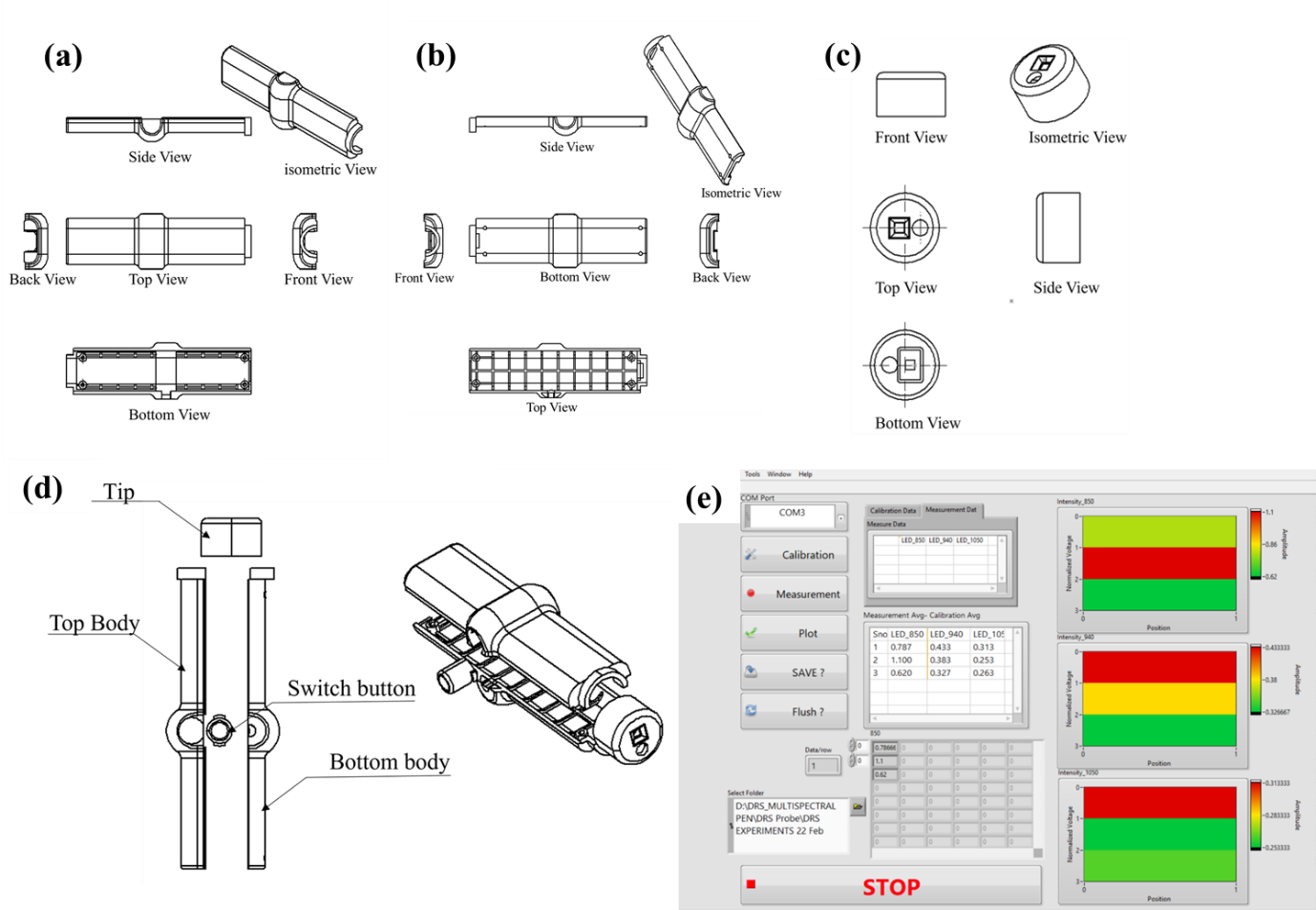

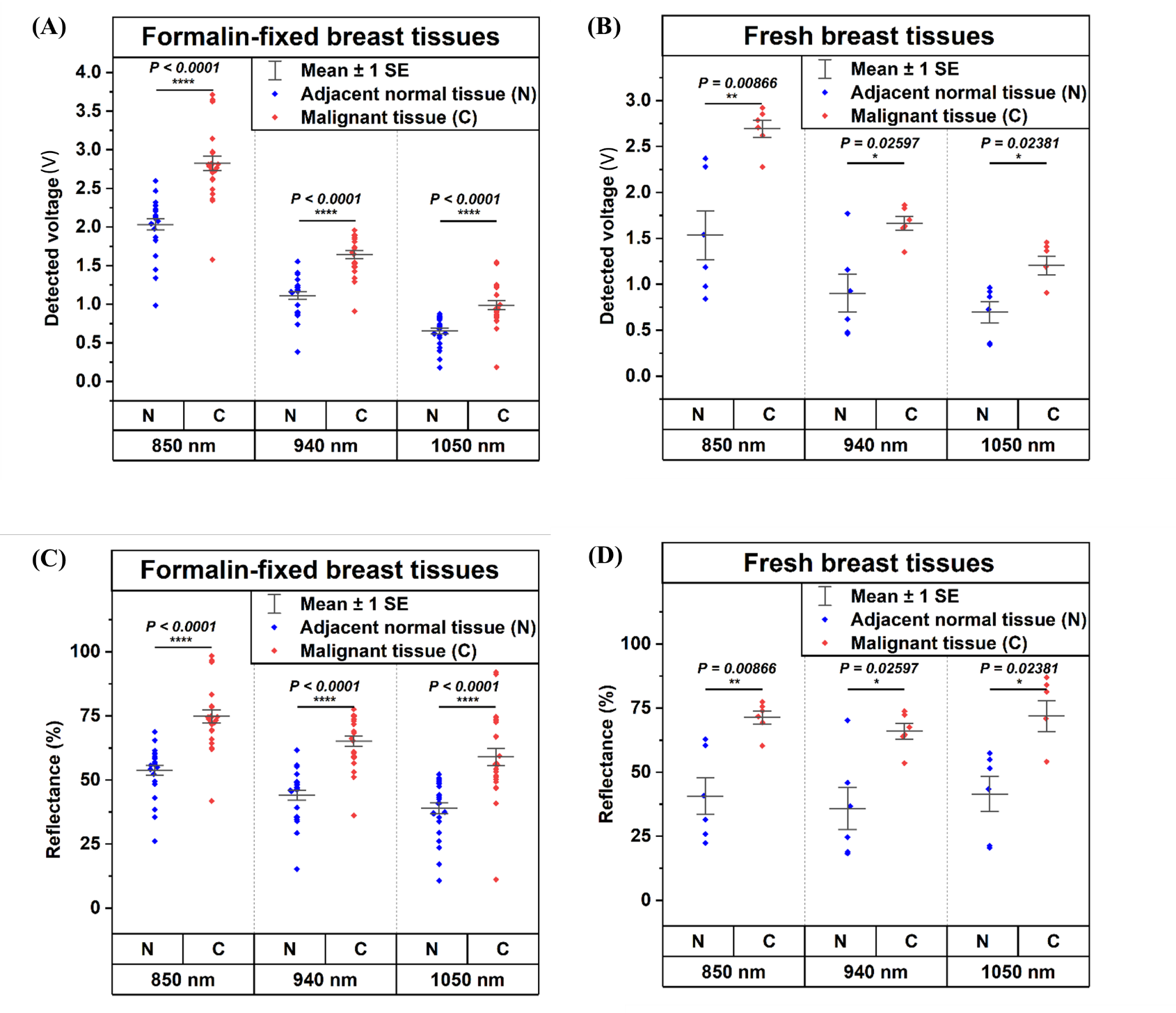


**Fig. S2:** Detected voltage (V) of (A) Formalin-fixed tissue samples and (B) Fresh tissue samples obtained at 850 nm, 940 nm, and 1050 nm, respectively.


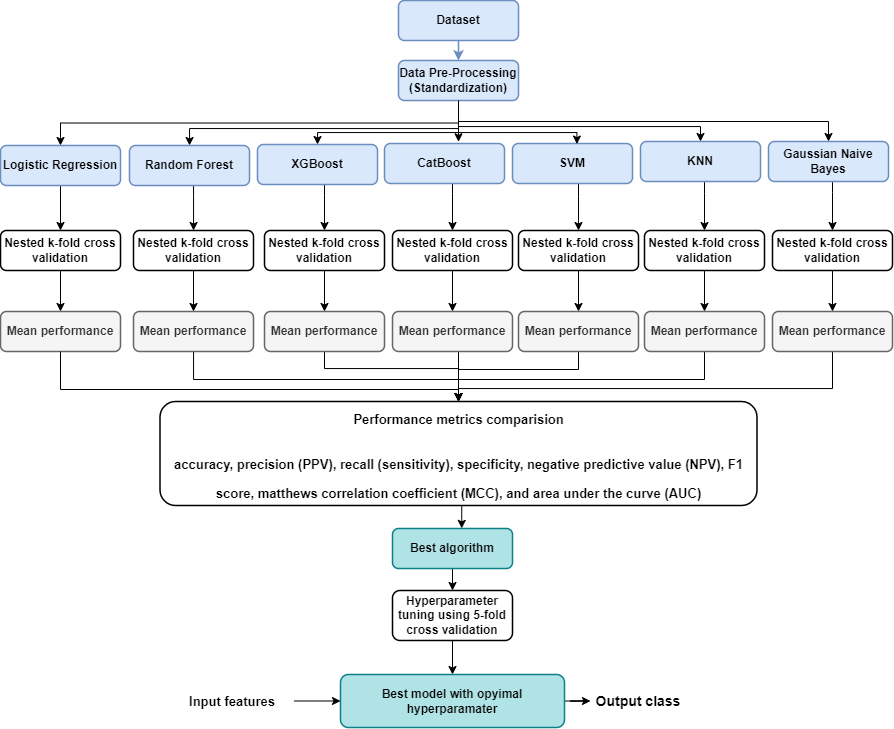


**Fig. S3:** Process flow chart of the algorithm and model selection.


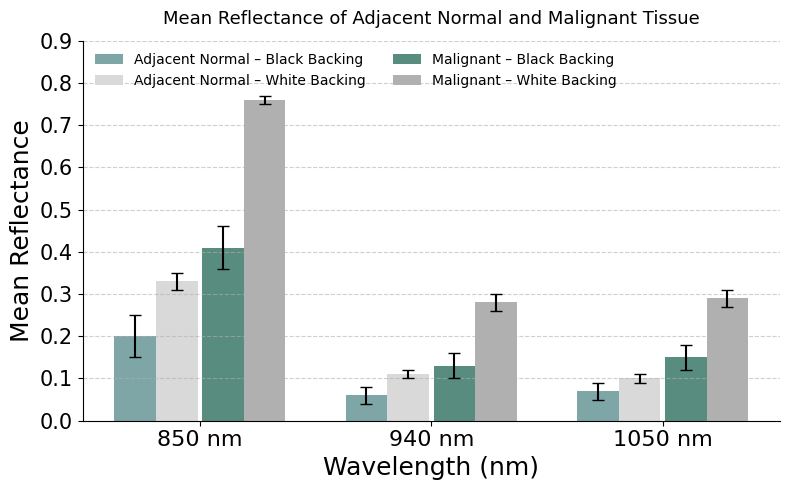

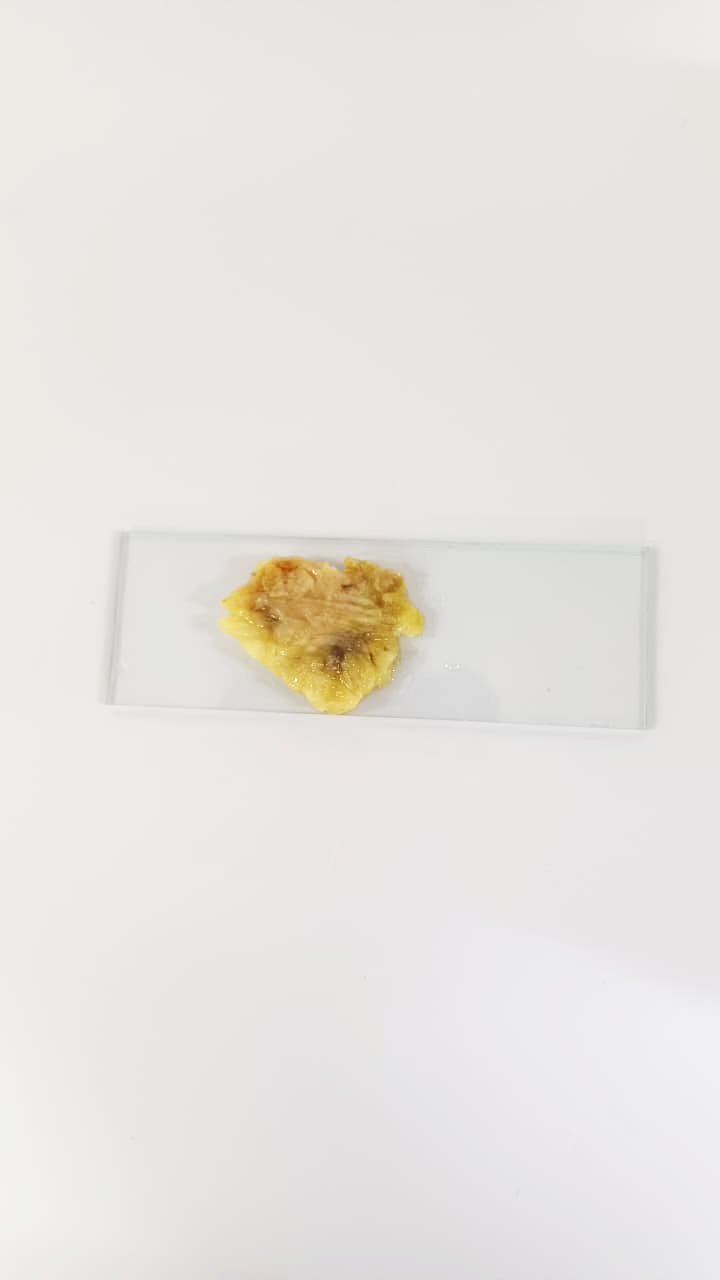

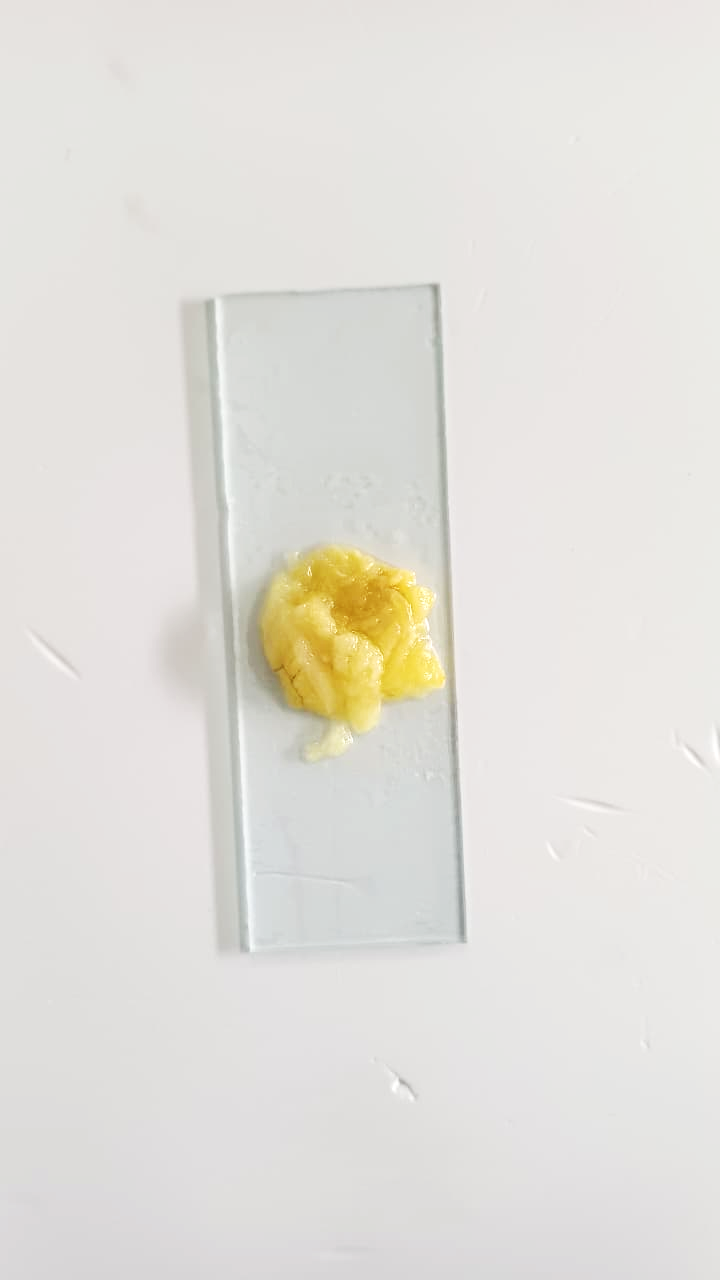


**(A)**

**(B)**

**(C)**

**(D)**

**(E)**

**Fig. S4:** Adjacent normal tissue measurements with: (A) Black backing, (B) White backing. Malignant tissue measurements with: (C) Black backing, (D) White backing, and (E) Bar plot of comparison of mean reflectance values with black and white backing for adjacent normal and malignant tissue samples obtained at 850 nm, 940 nm, and 1050 nm, respectively.


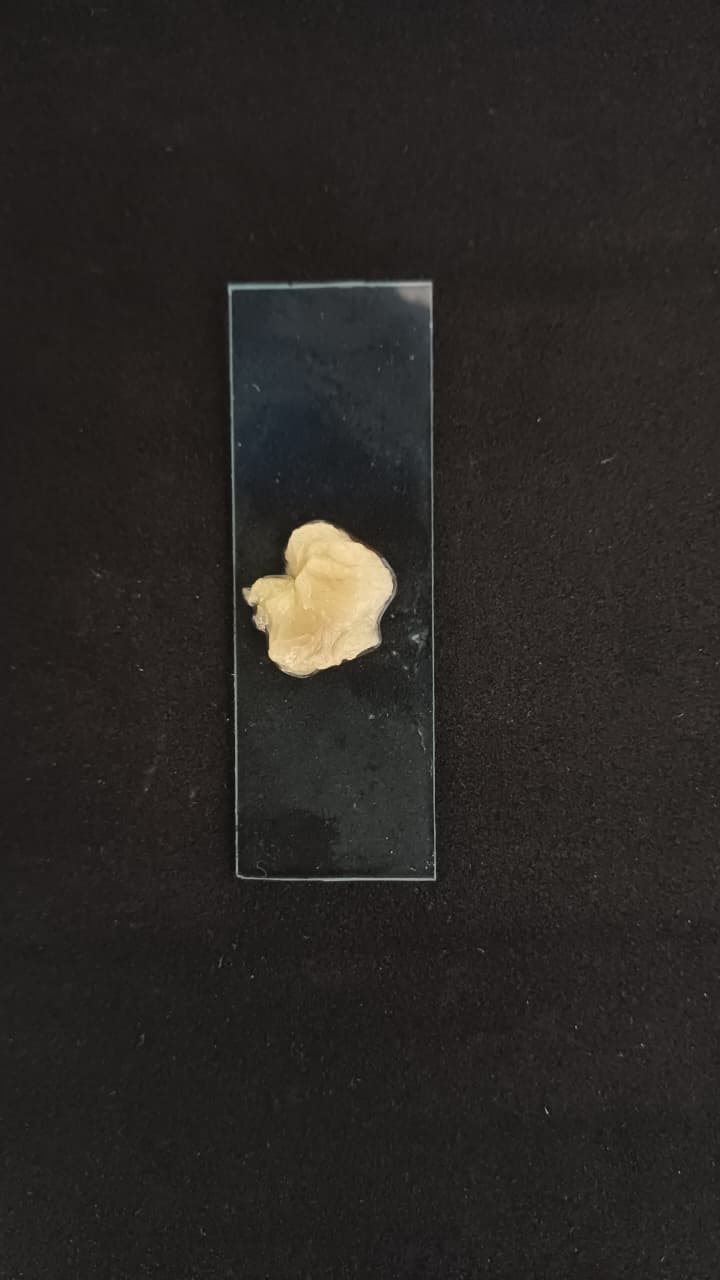

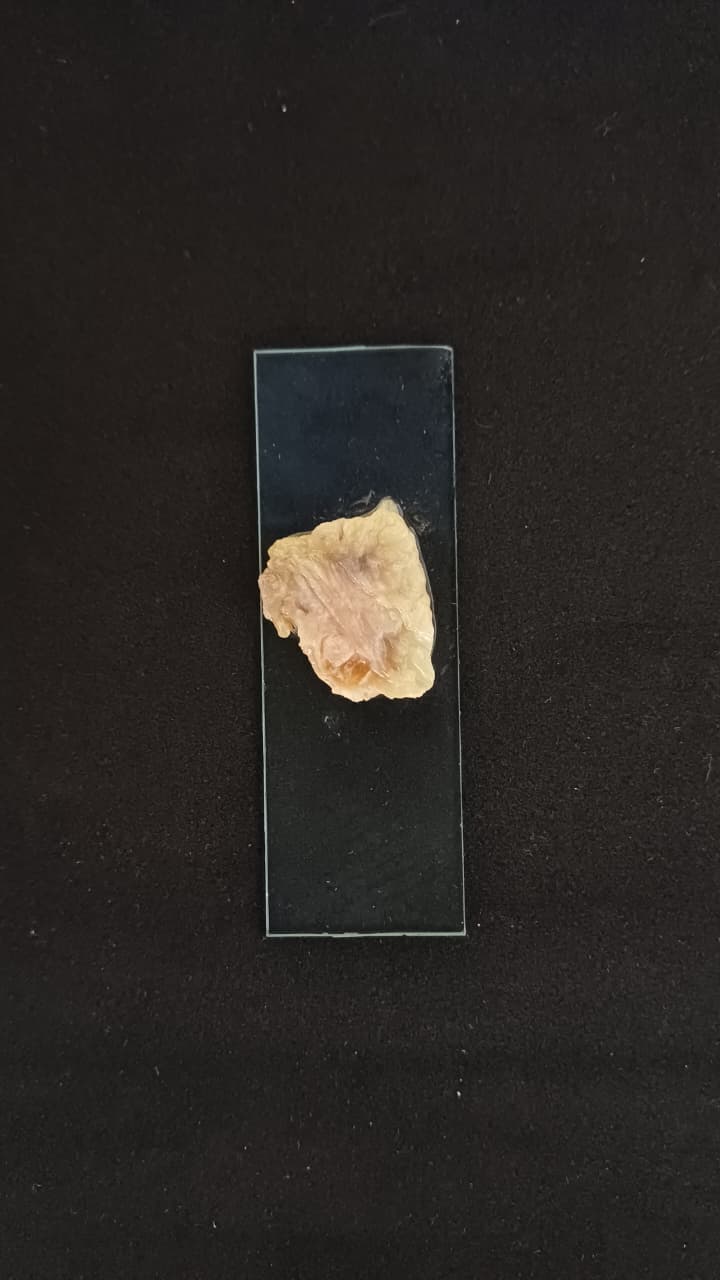

Supplement: Supplementary file 1 — Supplementary Material 1 [file 41598_2025_25867_MOESM1_ESM.docx]
